# Spatio-temporal trends in richness and persistence of bacterial communities in decline-phase water vole populations

**DOI:** 10.1038/s41598-020-66107-5

**Published:** 2020-06-11

**Authors:** Petra Villette, Eve Afonso, Geoffroy Couval, Aurélien Levret, Maxime Galan, Anne-Claude Goydadin, Jean-François Cosson, Patrick Giraudoux

**Affiliations:** 1 Université de Bourgogne Franche-Comté, CNRS UMR 6249 Chrono-environnement, Besançon, 25030 France; 2 Fédération Régional de Défense contre Organismes Nuisibles de Bourgogne Franche-Comté, Besançon, 25480 France; 3 CBGP, INRAE, CIRAD, IRD, Institut Agro, Université de Montpellier, Montpellier, France; 4 INRA, UMR BIPAR, Maisons-Alfort, 94700 France; 5 Institut Universitaire de France, Paris, 75231 France

**Keywords:** Next-generation sequencing, Microbial ecology, Population dynamics, Bacteria, Microbial communities, Parasitology, Pathogens

## Abstract

Understanding the driving forces that control vole population dynamics requires identifying bacterial parasites hosted by the voles and describing their dynamics at the community level. To this end, we used high-throughput DNA sequencing to identify bacterial parasites in cyclic populations of montane water voles that exhibited a population outbreak and decline in 2014–2018. An unexpectedly large number of 155 Operational Taxonomic Units (OTUs) representing at least 13 genera in 11 families was detected. Individual bacterial richness was higher during declines, and vole body condition was lower. Richness as estimated by Chao2 at the local population scale did not exhibit clear seasonal or cycle phase-related patterns, but at the vole meta-population scale, exhibited seasonal and phase-related patterns. Moreover, bacterial OTUs that were detected in the low density phase were geographically widespread and detected earlier in the outbreak; some were associated with each other. Our results demonstrate the complexity of bacterial community patterns with regard to host density variations, and indicate that investigations about how parasites interact with host populations must be conducted at several temporal and spatial scales: multiple times per year over multiple years, and at both local and long-distance dispersal scales for the host(s) under consideration.

## Introduction

Rodents are the most diverse mammal group in the world, with an estimated 2,277 species^1^, a global distribution that includes every continent, except Antarctica, and terrestrial habitats ranging from arid deserts to swamps and rainforest^2^. Generally characterized by fast life histories and large reproductive capacities, rodents are keystone species or ecosystem engineers in many of the ecosystems in which they are found^3,4^, but also frequently come into conflict with humans, causing significant loses in agricultural systems globally (see Singleton *et al.*^5^ for an overview). They also act as reservoirs for many socially and economically burdensome zoonoses (e.g.^6,7^), a pattern which is predicted to increase^8^.

Of particular interest to population ecologists for more than 100 years is the phenomenon of the rodent outbreak, in which the population in question exhibits dramatic multi-annual fluctuations in abundance, often with such a degree of regularity that these outbreaks are considered cycles, with four distinct phases. The increase phase is defined as a period of large increase in abundance from one spring to the next, and is followed by the peak phase, in which abundance is stable but high^9^. The peak phase can range in duration from mere weeks to a year, and is followed by the decline phase, in which abundance drops over as brief a period as months but can also take up to two years. The following low phase is characterized by stable but low population abundance.

The study of these systems has generated a large body of research^10^, but a consensus regarding the underlying demographic mechanisms of these outbreaks and their intrinsic or extrinsic drivers has yet to emerge (see^11^ for an overview). In addition, only a small subset of this research has considered the parasites hosted by these populations, despite diseases being the first hypothesis put forward to explain the decline phase^12^. This is in part due to the general conclusions drawn by Chitty in 1954^13^ after earlier research into *Toxoplasma* and *Mycobacterium tuberculosis* in cyclic field vole populations in the UK failed to find evidence that these parasites contributed to declines^13,14^: Chitty concluded that diseases were not important, and parasites and diseases in cyclic populations remained relatively understudied for decades.

Those early studies limited their investigations to a single infectious agent, but more recent investigations into parasites or disease as a driver of the decline have considered a wider variety of infectious agents. For example, Soveri *et al*.^15^ examined *Myodes glareolus* and *Microtus agrestis* collected from declining populations for a range of pathogens and histopathological changes, and reported widespread infection by *Taenia mustelae* and *Bordetella bronchiseptica*, increases in lymphoid tissue in the lungs, and enteritis. Cavanagh *et al*.^16^ estimated prevalences of cowpox virus and clinical signs of *Mycobacterium microti* infection in cyclic populations of *Microtus agrestis* in England, and found that both exhibited delayed density dependence with a lag of three to six months. Nicklasson *et al*.^17^ also detected delayed density dependence when they sampled a cyclic *Myodes glareolus* population in Sweden for Puumala virus. These studies have looked for correlations between disease prevalence and cycle phase, but there have been some experimental manipulations as well: Pedersen and Greives^18^ found that only a combination of both food addition and anti-helminth treatments prevented population declines in mixed *Peromyscus leucopus* - *Peromyscus maniculatus* populations that cycle in response to oak acorn masts. The unifying themes to these investigations have been either the explicit search for a decline-inducing disease, or the identification of extrinsic and intrinsic factors that determine risk of infection by, or prevalence of, one or a few parasite species.

What is less well understood is the role the parasite community may play as a driver of the decline, and conversely, the effects outbreaks have on parasite communities. Basic characteristics of parasite communities like richness, at the individual host or the host population scale, are predicted to depend on extrinsic factors like host abundance^19^, but the frameworks under which such predictions have been made often assume host populations are at equilibrium, and how parasite communities behave under dynamic host abundance is less well known. When considering just the bacterial component of the parasite community, it is becoming clear that parasite communities are not random assemblages but in fact structured by a variety of mechanisms including inter-specific interactions (e.g.^20,21^). Thus, the early studies that focused on one or a few bacterial parasites were likely missing important inter-specific interactions that contributed to the patterns observed. Technological constraints were a legitimate limitation to addressing community-based bacterial parasite questions in the past; culture methods, targeted DNA amplification, and the use of antibodies to detect parasites necessarily limit the scope of the investigation to either a pre-determined set of diseases or micro-organisms that are amenable to culturing (e.g.^16,17,22^). But the development of methods like high-throughput sequencing, that allow for the detection of a wide range of parasites that may not be culturable, enable us to ask more community-focused questions.

To lay groundwork for understanding how parasite communities change in fluctuating host populations, we considered the bacterial parasite community of the fossorial form of the water vole *Arvicola terrestris* in the Doubs and Jura departments of Franche-Comté, eastern France. In this region, *A*. *terrestris* are found primarily in grasslands ≥400 m above sea level^23^, and population densities fluctuate dramatically, peaking at over 200 individuals/ha every 5–8 years (mean period, 6 years) with concomitant forage production losses of over 1000 kg/ha/year^24^. Such peaks are followed by a decline phase which can be quite rapid, with a catastrophic decline in abundance during the winter months after the peak and a lack of recovery in population abundance the following spring.

We focus on bacterial parasites in part because other types of parasites have already received some attention: *A*. *terrestris* hosts a variety of helminths in this region, including the cestodes *Echinococcus multilocularis*^25^, *Taenia taeniaformis*^26^, *Taenia tenuicollis*, *Taenia crassiceps*, *Anoplocephala dentata*,* Paranoplocephaloides omphalodes*, and *Aprostatandrya* sp.^27^ and the nematodes *Trichuris arviclae*^28^, *Syphacia nigeriana*, *Trichuris muris*, *Capillaria murissilvatici*, and *Heligmosomoides laevis*^27^. Several viruses have also been detected here, including Puumala virus, lymphocytic choriomeningitis virus, and cowpox virus^22^. Like the early studies on parasites in cyclic rodent populations, research on these parasites has focused on linking patterns of prevalence or distribution to phases of the cycle: *Trichuris arvicolae* is most prevalent in increasing and peak populations and absent from declining populations^28^, while *Taenia taeniaformis* prevalence is higher at low local (<0.1 km^2^) host abundance than during outbreaks^26^. Cowpox prevalence is higher in late-peak populations than in early-peak and increasing populations, and seroprevalence is spatially aggregated, with some study sites featuring high (>70%) seroprevalences while in others, cowpox antibodies are entirely absent^22^.

While some of these studies do address co-infections (e.g.^22,27^), they are limited to parasites of the same kingdom (just viruses or just helminths). However, interactions between parasites are not restricted to within kingdoms or even within domains. For example, Joles *et al*.^29^ reported a strong negative association between bovine tuberculosis (bacteria) and gastrointestinal worms (helminths) in African buffalo, observing that co-infections are rare because co-infected animals experience accelerated mortality relative to animals infected with only TB or worms. Similarly, retrospective analyses of whooping cough (bacteria) and measles (virus) infections in children revealed interference between the two parasites, with infection by one removing the host from the susceptible pool of the other via mortality and subsequently influencing infection dynamics^30^. More broadly, microbial communities are coming to be seen as critical variables in the study of infection resistance^31^ and the evolution of multicellular organisms^32^, and communities in general are expected to influence ecosystem productivity under fluctuating conditions^33^. Identifying the bacterial parasites hosted by *A*. *terrestris* in this region is therefore crucial if we are to fully understand parasite community dynamics as a whole in this host, as well as the dynamics of the other parasites already observed.

We ask the following questions: does parasite community richness change over the course of an outbreak, and if yes, how? In addition to abundance, host populations are expected to exhibit changes in age structure, body condition, and reproductive output over the course of the outbreak, and we predicted that parasite richness at multiple scales would change concurrently with one or more of these factors. Larger populations are expected to host more parasites in much the same way that islands are expected to host more species under island biogeography theory^34^, and intrinsic host characteristics like age or body condition are likely to influence susceptibility to infection. Thus, we predicted that we would observe the demographic changes found in other cyclic species (an aging of the population^35^ and decline in body condition^36,37^ in the peak phase), and that richness would increase concurrently with increases in abundance or age structure, or declines in body condition. We also ask about the composition of the parasite community: which parasites remain at the end of the outbreak? Do they tend to co-occur in the same hosts or do they appear to infect animals independently? Are they prevalent or rare? We might expect that parasites found at the end of the outbreak when host abundance has declined tend to co-occur, reflecting facilitation effects that contribute to their persistence in the host population. We might also expect them to be relatively prevalent, indicative of their low pathogenicity and relative resistance to island effects.

## Methods

### Trapping and dissection of *Arvicola terrestris*

**Figure 1:**
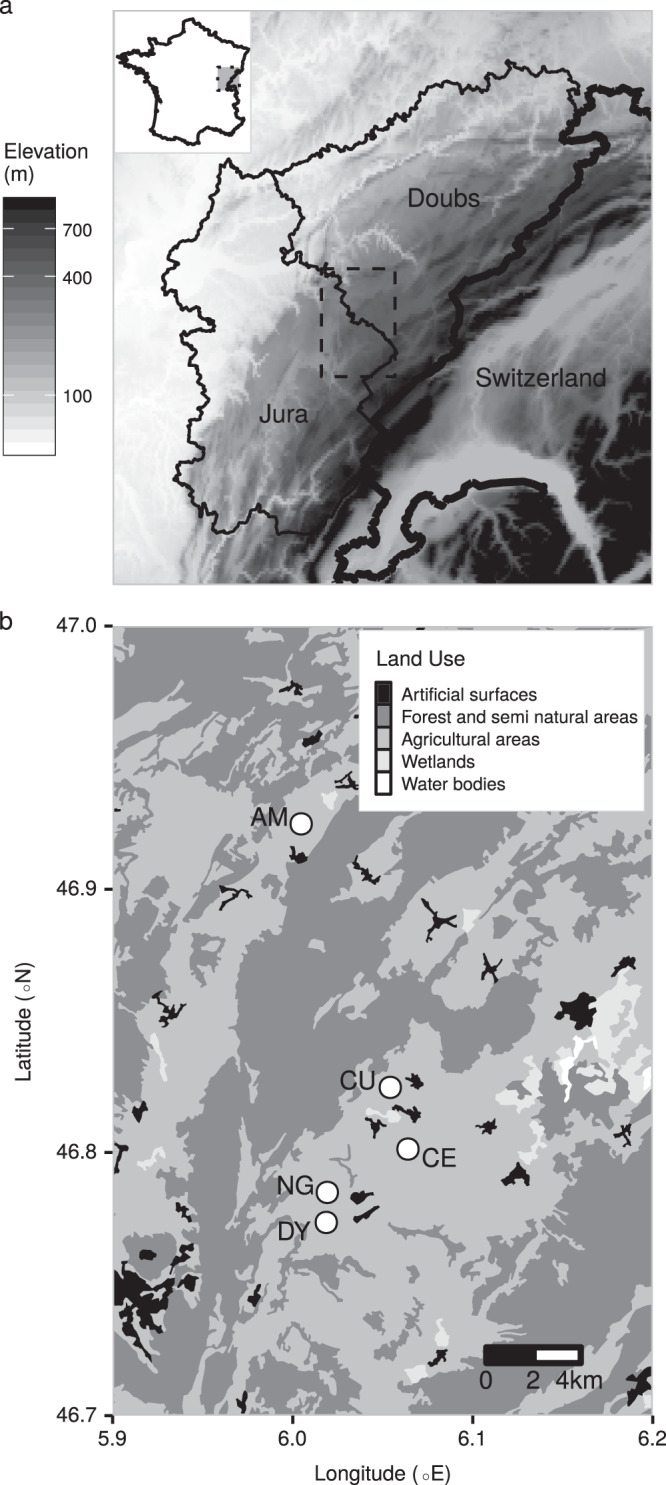
Maps of the study area in eastern France. (**a**) Elevation map of the Doubs and Jura Departments of eastern France, the region in which the study was carried out. Note that elevation has been log-transformed for clarity. GTOPO30 data courtesy of the U.S. Geological Survey, https://usgs.gov. Border data ©OpenStreetMap contributors, available under the Open Database License from www.openstreetmap.org via www.data.gouv.fr. (**b**) Land cover map of the study area with the 5 study sites Arc-sous-Montenot (AM), Censeau (CE), Cuvier (CU), Doye (DY), and Onglières (NG). Land cover categories shown are the upper-most level of the hierarchical nomenclature used in the CORINE Land Cover 2012 survey. In this figure, the land categories shown encompass the following sub-categories: (1) Artificial surfaces includes both continuous and discontinuous urban fabric, industrial or commercial units, and mineral extraction sites; (2) Agricultural areas includes pasture, complex cultivation patterns, non-irrigated arable land and land principally occupied by agriculture with significant areas of natural vegetation; (3) Forest and semi natural areas includes broad-leaved forest, coniferous forest, mixed forests, natural grasslands and transitional woodland-shrub; (4) Wetlands includes inland marshes and peat bogs; (5) Water bodies includes water bodies. Source: CORINE Land Cover 2012 survey (Version 20b2). Maps generated in R (version 3.5.2, R Core Team^55^), using packages egg^100^, ggplot2^101^, ggsn^102^, gtable^103^, raster^104^, rasterVis^105^, rgdal^106^ and sp^107^.

Populations were monitored in five study sites: Arc-sous-Montenot (AM, 6.00°E, 46.92°N), Censeau (CE, 6.06°E, 46.80°N), Cuvier (CU, 6.05°E, 46.82°N), Doye (DY, 6.02°E, 46.77°N) and Onglières (NG, 6.02°E, 46.78°N). These study sites are located in the Doubs and Jura departments of the Franche-Comté region of eastern France; this region is characterized by the Jura Massif, a mountain range bordered by a low-elevation plain (200–400 m above sea level) to the north west, and composed of a series of plateaus (400–700 m and 700–900 m in elevation) and the Jura mountains (900–1400 m in elevation). This region is a mix of managed forest, permanent grassland mowed for hay production, pasture in which cattle are grazed, and cultivated cereal parcels. Four of the sites (CE, CU, DY, and NG) are located in one zone of continuous grassland, centered around the village of Nozeroy in the Jura department (Fig. 1).The remaining site AM is 15 km north of NG in the Doubs department and separated from the other 4 sites by forest.

We conducted sampling sessions of 2 days duration in November of 2014 in AM and CE, at all five sites in April-May and November-December of 2015, 2016, 2017, and at all five sites in April-May of 2018. We set unbaited Sherman traps (H.B. Sherman Traps Inc., Florida, USA) between 8:30 and 9:30 in the morning, and checked them no more than 2 hours later, for a maximum of five checks during a day, after which we removed all traps. Traps were placed 50–60 m apart to avoid trapping the same burrow system twice and to ensure that sampling at high and low abundances was spatially similar. Two days of trapping at a site were sufficient for capturing at least 30 individuals when vole abundance was high, and this trapping effort was maintained in low-abundance periods though fewer individuals were captured. Concurrent with trapping, we also assessed local *A*. *terrestris* relative abundance using a transect method adapted from Giraudoux *et al.*^38^; 5 m-wide transects crossing the trapping areas were divided into intervals 10 m long and the proportion of intervals positive for fresh tumuli was taken as an index of abundance. Total transect length for a single sampling session ranged from to 1.5 to 1.8 km.

**Figure 2:**
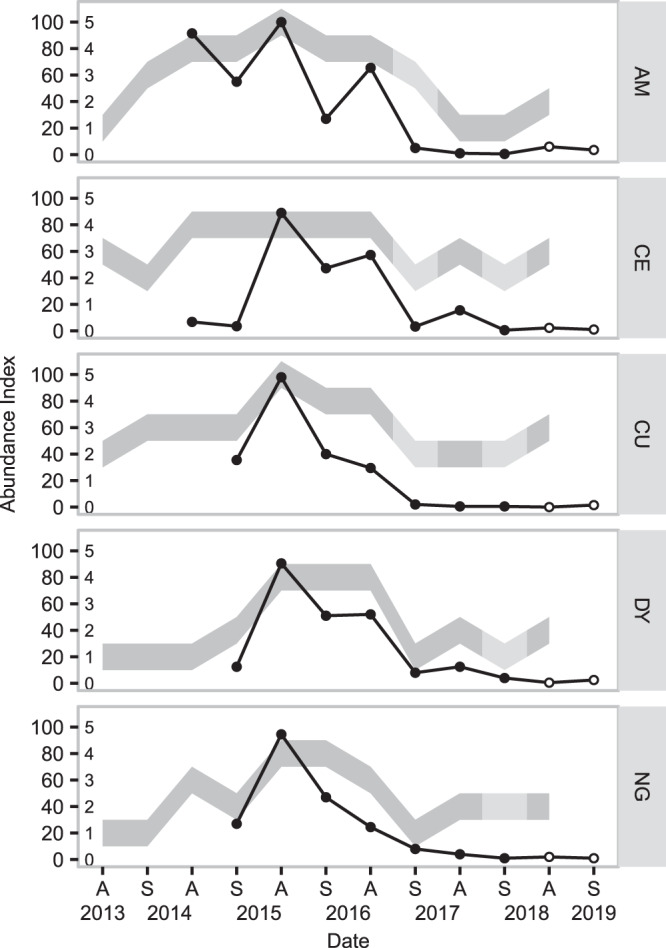
Abundance estimates for *A*. *terrestris* in five communes in Franche-Comté, France. Lines indicate changes in abundance indices estimated using walked line transects (longest transect, 1.2 km), while grey bands indicate changes in abundance indices based on visual assessments conducted at the commune level. Pale sections of the bands indicate approximated data; FREDON visual inspections were not conducted at these sites in these periods, and data shown are based on data collected from neighbouring communes (Villeneuve d’Amont for Arc-sous-Montenot and Supt for the other sites), calibrated using data collected from 2002–2016. Solid points indicate that animals were sampled when transects were walked; open symbols indicate that animals were not collected during these periods. Figure prepared in R (version 3.5.2, R Core Team^55^), using ggplot2^101^.

Abundance was also assessed at the commune-scale by technicians of the FREDON-BFC (Fédération Régional de Défense contre Organismes Nuisibles de Bourgogne Franche-Comté). Assessments were made in the spring and autumn but not in all studied communes every year (Fig. 2). The FREDON assessment uses a ranking system that ranges from 0 to 5: 0 - no *A*. *terrestris* sign in any parcel within the commune; 1 - low or no *A*. *terrestris* tumuli, voles and moles (*Talpa europaea*) cohabiting the same tunnel systems; 2 - *A*. *terrestris* tumuli present in some parcels within the commune, mole burrow systems still present in some parcels; 3 - *A*. *terrestris* tumuli present in some parcels within the commune, few or no mole burrow systems present in the commune; 4 - *A*. *terrestris* colonies established in the majority of meadows and within pastures; 5 - all of the commune is colonized by *A*. *terrestris*. The FREDON index does not directly translate to transect-based indices, partly because it is applied at the commune scale and not the parcel scale, but Giraudoux *et al*.^38^ have shown that levels 0–1 correspond to densities <100 animals/ha, level 2 to 100–200 animals/ha, and levels 3–5 to >200 animals/ha.

Trapped animals were dissected on site following Auffray *et al*.^39^. One at a time, individuals were euthanized via cervical dislocation and at least 0.5 ml of blood was collected via cardiac puncture with a syringe. For each animal, the heart and portions of the liver, lungs, kidneys, and spleen were stored in RNAlater and frozen within 36 hours of collection. Dissection tools were sterilized with bleach, water and alcohol between animals, but not between organs. We do not think that cross contamination between organs during the dissection process is significant because we have found that bacterial community composition differs significantly between organs collected from the same animal even when dissection tools are not sterilized between organs^40^.

Multi-annual abundance cycles in some rodents, including other populations of*Arvicola terrestris*, have been accompanied by changes in demographic parameters like age structure and reproductive output^35^ and physiological characteristics like body weight^36,37^. To determine if our populations under study also undergo changes in age structure, reproduction, and physiology, we collected additional measures and tissues from each animal. During dissection, animals were weighed and sexed, and reproductive characteristics were recorded: testicle position (scrotal or abdominal) and testes length and width for males, nipples (lactating or not lactating), vagina (open or closed), pubic symphysis (open, slightly open, or closed), and number of embryos or placental scars for females. Males were considered reproductively active if their testes were scrotal, and females were considered reproductively active if they carried embryos or were lactating.

To determine if our populations also exhibit changes in age structure over the course of the outbreak cycle, we used dry eye lens weight as a proxy for animal age. Eyes were removed and stored whole in 75% alcohol at 4 °C until they could be weighed; the crystalline lenses were removed from the eye tissue and allowed to dry at 45–55 °C for at least 5 days before being weighed to the nearest 1/10 mg. Lens weights hereafter refers to the pooled weights of the two lenses from each animal; in instances where only one lens could be weighed (the animal was missing an eye or the lens was destroyed during dissection), lens weight for that animal was simply double the weight of the single lens. In instances where the two lens weights differed by more than 50% of their mean (i.e. the smaller lens was less than one third the mass of the larger lens), the larger of the two lenses was doubled. To asses changes in age structure of the sampled populations through time, we used a two-way ANOVA with sampling date, sampling site, and an interaction term as factors followed by Tukey HSD tests to identify differences in lens weights in sampling sessions within sampling sites.

Body condition is used by wildlife ecologists as a proxy for a range of characteristics including nutritional status^41^, fat content^42,43^, and Darwinian fitness^44^, with the underlying assumption that animals that are heavier for their size are “healthier”. We used the residuals of weight regressed onto body length squared as a measure of body condition for all males and non-pregnant females. Changes in body condition of these two groups were assessed separately using linear modeling with sampling period, location, and an interaction term. Location was not a significant predictor of body condition for males and dropping it and the interaction term from the male model improved model fit as determined by AIC score, so location was not included for males. Tukey HSD tests were performed to detect changes in body condition; these pairwise comparison were performed within locations for non-pregnant females.

Animals were treated in accordance with European Union guidelines and legislation (Directive 86/609/EEC). The Chrono-environnement laboratory received approval from the Comité d’Ethique Bisontin en Expérimentation Animale (CEBEA N°58) for the sampling of rodents and the storage and use of their tissues. The rodent species investigated in this study does not have protected status (see IUCN and CITES lists), and is listed as a pest, subject to control, under Article L201-1 of the Code Rural et de la Pêche Maritime of French law.

### Detection of bacterial parasites

DNA extraction and PCR amplification of bacterial parasite DNA followed the lab protocol described in Villette *et al.*^40^ DNA extractions were conducted using the Qiagen DNeasy 96 Blood and Tissue kit (Qiagen); tissue samples of spleens from each animal were extracted individually, while tissue samples from the heart, liver, lungs, and kidney were pooled and extracted together for each animal (spleens were prepared separately to facilitate comparison to animals sampled in this region in 2003–2005^22^). Tissue samples were incubated overnight at 56 °C and processed following kit instructions except for the last step: two elutions of 100 *μ*l of warmed buffer AE were preformed, instead of 1 elution of 200 *μ*l of buffer. One well of each 96-well plate was used as a negative control (no starting material was used, but all other steps were performed). DNA concentrations of DNA extracts were measured using a NanoDrop 800 UV-Cis Spectrophotometer (Thermo Fischer Scientific, France); DNA concentrations were all greater than 5 ng/*μ*l.

PCR amplification of the V4 region of the 16S rRNA bacterial gene was conducted using a protocol derived from Kozich* et al.*^45^, using reagents and primers described in Galan *et al.*^46^; we used 16S-V4F/16S-V4R primers dual-indexed with 8bp-indices (i5 and i7) and Nextera Illumina adapters to perform two PCR replicates for each DNA sample. PCR was performed with 5 *μ*L of HotStarTaq Master Mix (Qiagen), 4 *μ*L of combined forward and reverse primers (2.5 *μ*M) and 2 *μ*L of DNA extract, for a final volume of 11 *μ*L for each PCR replicate. The PCR program consisted of an initial denaturation step at 95 °C for 15 minutes, followed by 40 cycles of denaturation at 95 °C for 20 s, annealing at 55 °C for 15 s, and elongation at 72 °C for 5 minutes, followed by a final extension step at 72 °C for 10 minutes. While high numbers of PCR cycles are known to increase chimera formation, this parameter is most critical in applications that involve the bulk amplification of tagged/indexed amplicons, for example when using the Illumina TrueSeq library preparation kit^47^. We believe that using separate amplicon libraries for each sample minimized this effect in our data, and while fewer PCR cycles would further reduce the risk of chimera formation, this would also reduce our ability to detect bacterial parasites, which are sometimes at very low quantities in animal tissues.

We used a QIAxcel DNA Screening kit on the QIAxcel platform (Qiagen) to confirm PCR amplification. Negative controls (PCR reagents but no DNA) were included in each PCR plate, and positive controls were included in each set of 9 PCR plates. DNA used for positive controls was from *Bartonella taylorii*, *Borrelia burgdorferi*, *Mycoplasma mycocoides*, *Mycoplasma putrefaciens*, and *Psedumonas aeruginosa*.

After PCR, indexed amplicons were pooled and purified using 1.25% agarose gel run for 2 hours at 100 V. The excised gel was cleaned up using the Qiaquick Gel extraction kit on the QiaCube automation platform (Qiagen), and purification was confirmed using a DNA High Resolution kit on the QIAxcel (Qiagen). Quantitative PCR to quantify the DNA of the final library was performed with the KAPA library quantification kit (KAPA Biosystems) as per^46^ prior to the library being loaded on the Illumina MiSeq flow cell using a 500 cycle reagent cartridge.

The resulting sequences were filtered, clustered and assigned taxonomic classification using the FROGS pipeline^48^; sequences with ambiguous bases were removed and sequences were filtered for length before duplicate sequences were merged. OTUs (Operational Taxonomic Units) or groups of similar sequences analogous to species, were formed using the Swarm algorithm^49^; two clustering iterations are performed - the first to denoise the data, and the second using the first iteration as seed sequences for grouping.

Chimera detection utilized VSEARCH^50^ with *de novo* UCHIME^51^, in conjunction with cross-validation; that is, a sequence was considered chimeric and removed only if it was flagged as chimeric in all samples in which it appeared. Following the above steps, taxonomic assignment was conducted using blastn+ and the SILVA 16S database version 132^52^: the best alignment hits were retained for each seed sequence, to a limit of 500, and were used to assign a consensus taxonomic affiliation. Blastn+ results for seed sequences, including best hits, % similarities, e-values, etc. can be found in Supplementary Table S13.

The resulting read-abundance table was further cleaned using the protocol recommended by Galan *et al*.^46^. Read abundances were filtered using a mis-assignment rate of 0.002%, and OTUs that were taxonomically assigned to genera that include known obligate parasites were selected for further analysis. OTUs that were assigned to genera that include opportunistic pathogens but not obligate parasites were not retained; for example, *Acinetobacter baumannnii* is an emerging nosocomial pathogen, causing serious infections in veterinary clinics^53^, but as infections appear to be primarily opportunistic, it is widespread in the environment, and doesn’t appear to have a reservoir host species^54^, OTUs assigned to *Acinetobacter* would not be retained for analysis. Chimera detection was performed again on the seed sequences of the selected OTUs using USEARCH v11.0.667 (high-confidence mode) with the SILVA 16S database as a reference database; OTUs with seed sequences identified as chimeras using this method were also removed from the analysis. Seed sequences of the OTUs presented here were submitted to GenBank (accession numbers MN594311 - MN594465, see Supplementary Table S12 for additional details).

### Richness

We adopt here a community ecology approach and use the terminology of Poulin and Morand^34^ with respect to scale; at the individual host scale we have the parasite infracommunity, or all of the parasites infecting one host. At the host population scale is the parasite component community, or all of the parasites infecting one host population. At the host meta-population scale we have the meta-community, or all of the parasites found within the host meta-population.

All statistical analyses were performed using R software (version 3.5.2, R Core Team^55^). Some of the specific packages and functions used are listed inline, the remainder are listed in the Supplementary Material. We calculated infracommunity richness for each animal by counting the number of unique OTUs found within each animal (i.e. if an OTU appeared in both the spleen and other organs of an animal, it was only counted once). We used generalized linear modeling with a negative binomial distribution and log link to determine if infracommunity richness varied between sampling periods (function glm.nb of package MASS^56^). We don’t attempt to relate infracommunity richness to other variables like body condition or abundance because these and other variables appear interrelated, and disentangling their relationships is beyond the scope of this manuscript.

Component community richness and meta-community richness was assessed in two steps; first, we constructed species accumulation curves using animals as our unit of effort and sampling without replacement. Animals were pooled by sampling session and location to visualize component community richness, and by sampling session (sites pooled) to visualize meta-community richness. Second, we used the iNEXT function of package iNext^57,58^ to extrapolate these curves to the largest sample size at the component and meta-component scales, 37 and 165 animals respectively. That is, we estimated the number of unique OTUs we would have found had we sampled 37 animals in each sampling session at each location, and 165 animals in each session when sites are pooled. The extrapolation performed by iNEXT requires an asymptotic estimate of species richness (the number of species or OTUs detected with an infinite sample size)^57^, we used the Chao2 richness estimator as it uses incidence data and has been found to be relatively precise and minimally biased compared to other estimators^59^. We chose to extrapolate curves only to the largest sample size because such extrapolations are expected to be unbiased only at sample sizes two to three times larger than the observed data^60^. We used 100 bootstrap iterations to generate 95% confidence intervals.

### OTU persistence

To assess the persistence of OTUs within the host meta-population over the course of the outbreak, we selected the OTUs found in animals in spring 2018, and performed co-occurrence analysis using the whole data set (not just occurrence data from spring 2018). We used the cooccur function in package cooccur^61^ to measure the associations between the isolated OTUs (i.e. are some pairs of OTUs found co-infecting an animal more often than we would expect if these OTUs are distributed randomly in the host population).

## Results

### Host population demographic characteristics and abundance

A total of 869 animals were captured, with an average of 23 animals per sampling session (range: 1 to 37, Table 1); the sex ratio (proportion of males) varied from 0.08 (2 males to 24 females) to 1.00 (1 male to no females) with a mean of 0.50. 845 of them were analyzed for their bacterial parasites.

**Table 1:** Sample sizes for each sampling session at each site.

Date	AM	CE	CU	DY	NG
Autumn 2014	26 (0.08)	31 (0.39)	0	0	0
Spring 2015	31 (0.48)	36 (0.69)*	37 (0.54)*	31 (0.52)	34 (0.56)
Autumn 2015	37 (0.57)*	37 (0.51)*	35 (0.23)	30 (0.43)	36 (0.44)
Spring 2016	30 (0.33)	31 (0.55)	37 (0.43)	32 (0.47)	32 (0.56)
Autumn 2016	33 (0.36)	33 (0.33)	33 (0.52)	37 (0.3)*	30 (0.57)
Spring 2017	4 (0.5)	14 (0.64)	1 (1)	22 (0.5)	11 (0.36)*
Autumn 2017	7 (0.43)	34 (0.5)	9 (0.56)	16 (0.31)	5 (0.2)
Spring 2018	2 (1)	4 (0.5)	1 (1)	5 (0.4)	5 (0.8)

*Not all animals from these sampling sessions were analyzed for their bacterial parasites. Sample sizes for parasite analysis are as follows: CE spring 2015 - 33; CU spring 2015 - 36; AM autumn 2015 - 32; CE autumn 2015 - 32; DY autumn 2016 - 32; NG spring 2017 - 6. Table prepared in R (version 3.5.2, R Core Team^55^) and exported with packaged xtable^108^.

Relative local abundance for each sampling session, as assessed by transect indices, ranged from 0.5% to 100% (Fig. 2) while commune abundance as assessed by the FREDON during this period ranged from 1 to 5 and followed similar trends to our transect-based abundance indices, with the exception of Censeau in autumn 2014 and spring 2015. Based on abundance indices at the two scales, we identified 2014–2015 as the high or peak phase, 2016 as the decline phase, and 2017–2018 as the low phase.

**Figure 3:**
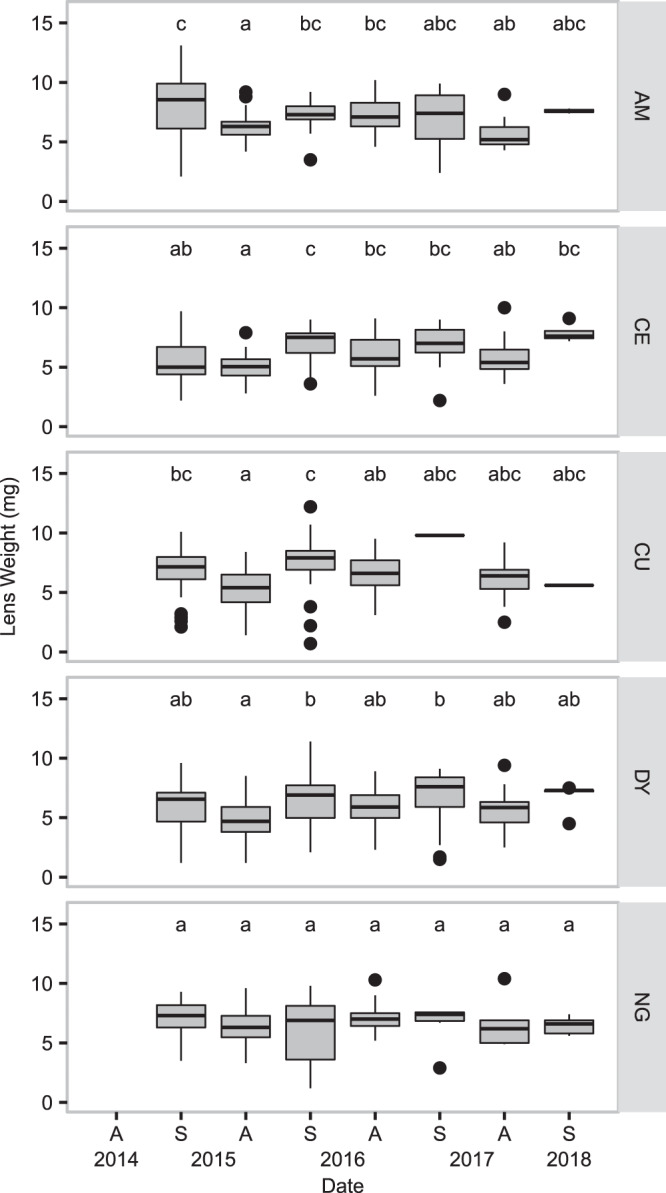
Age structure of *Arvicola terrestris* populations. Box and whisker plots of dry eye lens weights for males and females collected in five sites (AM, CE, CU, DY, and NG) from spring 2015 to spring 2018 (eye lenses were not collected in autumn 2014). Letters indicate groups of populations that do not differ significantly; comparisons were restricted to within locations. Figure prepared in R (version 3.5.2, R Core Team^55^), using ggplot2^101^.

Mean dry lens weight for sampling sessions ranged from 4.8 mg to 9.8 mg, and while we did detect variation in lens weight over the course of the sampling period, we did not detect a multi-annual trend of aging. Differences in age structure of populations tended to be seasonal, with older populations detected in the spring and younger populations in the autumn (Fig. 3, Kruskal-Wallis rank sum test, df = 6, *χ*^2^ = 79.05, p < 0.001).

**Figure 4:**
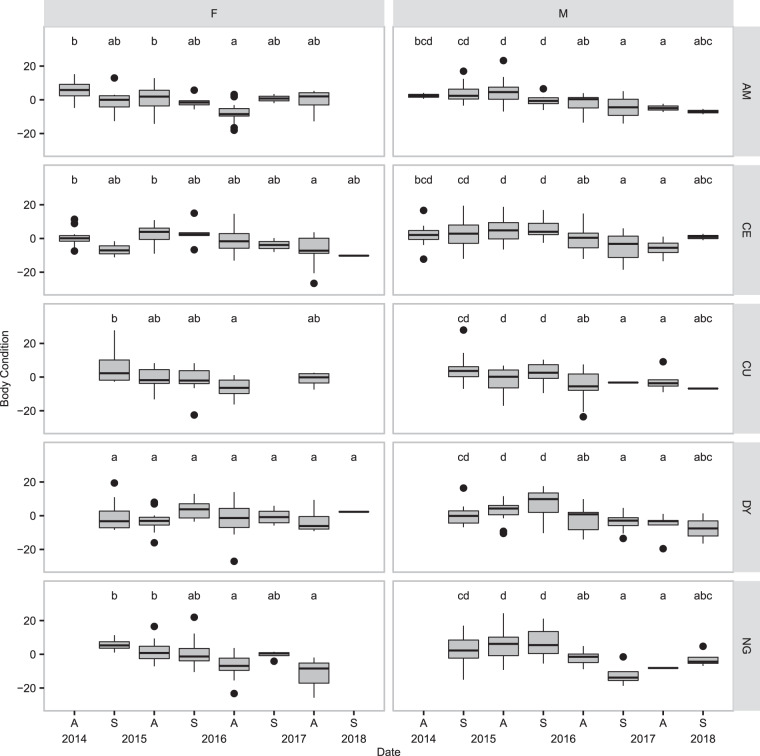
Body condition of non-pregnant females (F) and males (M). Box and whisker plots of body condition of males and non-pregnant females in five sites (AM, CE, CU, DY, and NG) from autumn 2014 to spring 2018. Body condition was estimated as the residuals of weight (in grams) regressed onto length from nose to anus squared. Letters indicate groups of populations that do not differ significantly, as determined by comparisons of confidence intervals of estimated marginal means following linear modeling. Location was not a significant predictor of body condition in males, therefore males collected in the same period but different locations were pooled together. Location and a date-location interaction term were significant when modeling females, therefore comparisons are restricted to sampling periods within locations. Figure prepared in R (version 3.5.2, R Core Team^55^), using ggplot2^101^.

Body condition did however decline markedly in males over the course of the outbreak; males collected in autumn 2016 and spring and autumn 2017 (decline and low phase) had significantly lower body condition than males collected in spring and autumn 2015 and spring 2016 (high and early decline phase, Fig. 4b, ANOVA, F_7,390_ = 15.95, p < 0.001). Female body condition also declined over the course of the outbreak, but not in all locations, and exhibited site-specific and seasonal effects (Fig. 4a, ANOVA, F_32,320_ = 3.54, p < 0.001). Autumn body condition in AM and NG is lower in 2016 than in previous years, but in AM recovery can be observed the following year, while in NG body condition remains low. In CE, the decline in autumn body condition doesn’t become significant until 2017, the low phase, and in CU and DY, we did not detect significant changes in autumn body condition at all. In all five locations, spring body condition is stable over the sampling period. Mean body condition of non-pregnant females and the proportion of females that had reproduced (pregnant, lactating, or with visible placental scars) within sampling sessions was weakly positively correlated (Pearson correlation coefficient = 0.36, t = −2.2, p = 0.035).

**Figure 5:**
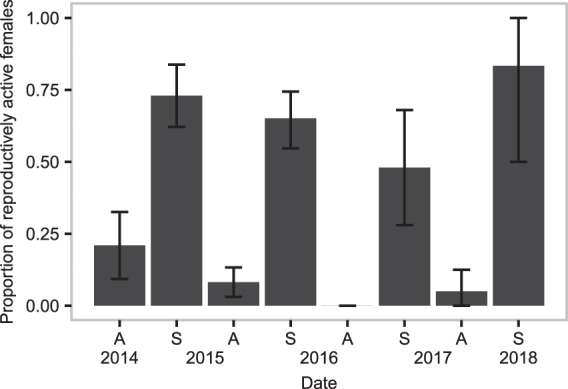
Reproduction in female *Arvicola terrestris*. Proportion of females that were reproductively active for each sampling period; all five sampling locations were pooled together. Females were classed as reproductively active if they were pregnant or lactating. Error bars are bootstrapped 95% confidence intervals. Figure prepared in R (version 3.5.2, R Core Team^55^), using ggplot2^101^.

Females were reproductively active (pregnant or lactating) primarily in the spring, with the proportion of females reproductively active ranging from 50% to 83% (Fig. 5); female reproduction was lowest in spring 2017 (the low phase) relative to spring 2015 (high phase), while spring 2016 and 2018 reproduction did not differ significantly from spring 2015 (binomial GLM with logit link, odds-ratios and 95% confidence intervals: spring 2016 = 0.52, 0.24–1.08; spring 2017 = 0.32, 0.11–0.89; spring 2018 = 1.32, 0.19–26.39). The number of embryos found in pregnant females ranged from 1 to 8 (mean 4.91) but did not vary between springs (Poisson GLM with log link, *χ*^2^ = −3.44, p = 0.33).

### Bacterial parasites

**Figure 6:**
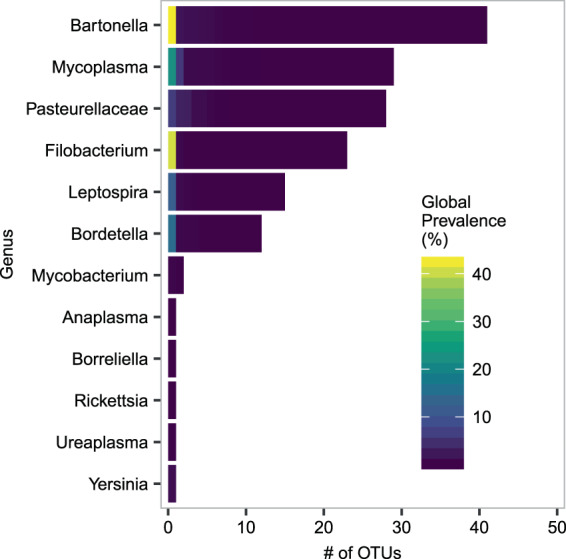
Bacterial parasite OTU taxonomic affiliations and global prevalences. The number of bacterial parasite OTUs obtained from *A*. *terrestris* populations in Franche-Comté, grouped by the bacterial taxon to which they were assigned. Bars are stacked tiles coloured to indicate global prevalence of each OTU; for example, we detected 23 OTUs assigned to *Filobacterium*, 22 of them had prevalences of less than 10% and appear dark, while one had a global prevalence of greater than 40% and is therefore coloured yellow. 27 of the 28 OTUs assigned to the family *Pasteurellaceae* could not be assigned to a lower taxonomic level, therefore all 28 are grouped as *Pasteurellaceae*. Figure prepared in R (version 3.5.2, R Core Team^55^), using ggplot2^101^.

We retained a total of 155 OTUs representing at least 13 genera in 11 families; OTU taxonomy and prevalence is shown in Fig. 6. One hundred twenty eight of these OTUs could be assigned to genera; the remaining 27 could not be taxonomically resolved below the level of the family *Pasteurellaceae*. The most common genera were *Bartonella*, *Mycoplasma*, *Filobacterium*, *Leptospira* and *Bordetella*; together these genera accounted for 77% of the parasitic OTUs in the data set. Global prevalence of individual OTUs ranged from 0.1% to 42.5% (*Bartonella*) with the majority of OTUs (137) found at a prevalence of less than 1%. Raw data from the eight Illumina MiSeq runs were deposited in the Zenodo data repository (10.5281/zenodo.3784637).

### Bacterial parasite richness

**Figure 7:**
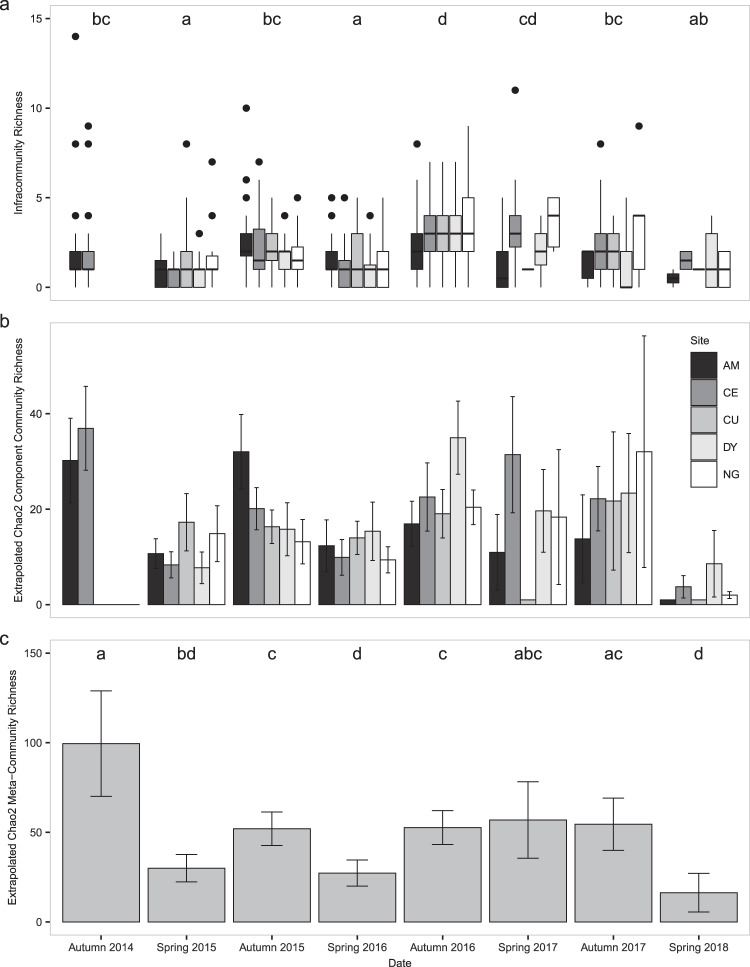
Bacterial parasite community richness. (**A**) Box plots (box and whisker diagrams) of bacterial infracommunity richness (OTUs/animal) in *Arvicola terrestris* collected from five locations in Franche-Comté from autumn 2014 to spring 2018. Letters indicate significant differences between dates. (**B**) Extrapolated Chao2 component community richness estimates (OTUs/population). Samples sizes were extrapolated to 37 animals/session. (**C**) Extrapolated Chao2 meta-community richness (OTUs/sampling period). Sample sizes were extrapolated to 165 animals/session. Error bars are 95% confidence intervals. Letters indicate non-overlapping confidence intervals. Figure prepared in R (version 3.5.2, R Core Team^55^), using packages egg^100^ and ggplot2^101^.

Infracommunity richness exhibited seasonal patterns and phase-related patterns: richness tended to be higher in the autumns than in springs, with the exception of spring 2017 in which richness was similar to autumns and differed significantly from the other spring sampling periods (Fig. 7a, negative binomial GLM with log link, *χ*^2^ = 161.03, p < 0.0001). Autumn richness also exhibited a peaked in autumn 2016.

**Figure 8:**
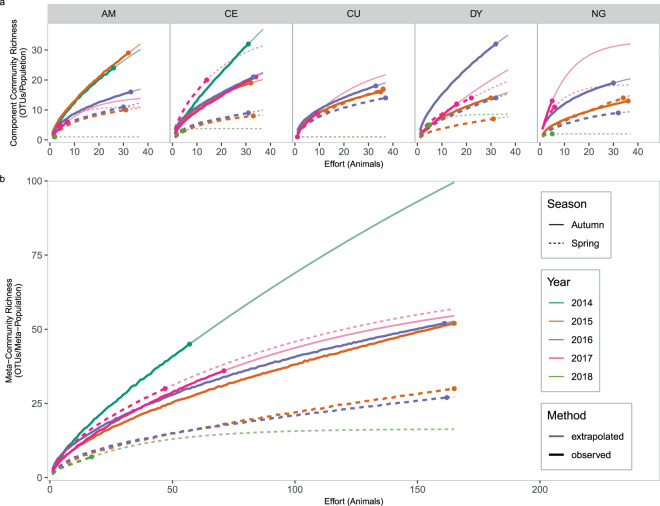
Species accumulation curves. (**A**) Species accumulation curves for component communities (animals pooled within sampling sites) and (**B**) meta-communities (animals from different sampling sites pooled together within dates). Heavy lines indicate portions of the curve constructed by sampling observed data with replacement, points indicate the observed number of OTUs, and the fine lines are extrapolated curves using the Chao2 estimator. Figure prepared in R (version 3.5.2, R Core Team^55^) using packages egg^100^ and ggplot2^101^.

Raw component community richness varied from 1 to 32 unique OTUs per sampling session, but this is clearly partly due to sampling effort. Species accumulation curves (Fig. 8a) clearly show that spring component community richness increases the fastest in 2017 (low phase) in 3 of the 5 sites, the exceptions being CU and AM, in part due to few animals being collected in this period. Autumn species accumulation curves are less clear, with the steepest curve occurring in 2014 in CE, 2015 in AM, 2016 in CU and DY, and 2017 in NG. However, extrapolated component community richness does not indicate that spring 2017 communities were richer than other springs for any site except Censeau, based on 95% confidence intervals (Fig. 7b). There also does not appear to be a strong seasonal pattern in extrapolated component community richness, or consistency between locations.

Raw meta-community richness varied from 7 to 52 OTUs/meta-community, and like component community richness, this is somewhat related to sampling effort. Species accumulation curves (Fig. 8b) clearly show that the meta-community richness of spring 2017 (low phase) increased faster than other springs, while autumn 2014 (high phase) has the steepest autumn meta-community curve despite a smaller sample size both in terms of number of animals collected and sites visited. Extrapolated Chao2 richness estimates at the meta-community scale exhibit broadly similar patterns to species accumulation curves; we see high Chao2 richness estimates in the early peak phase (autumn 2014), with subsequent autumns having less rich communities until autumn 2017 (Fig. 7c). Spring extrapolated richness is lower than autumn richness for all years except 2017 when spring richness is similar to autumn richness. This appears to be somewhat related to infracommunity richness; when sampling locations were pooled together, spring infracommunity richness was higher in 2017 than in either 2015 and 2016, and autumn animals hosted richer infracommunities than spring animals except for spring 2017 (as high as the autumn periods) and autumn 2017 and 2014 (as low as the spring periods, negative binomial GLM, *χ*^2^ = 145.64, p < 0.0001).

### OTU persistence

**Figure 9:**
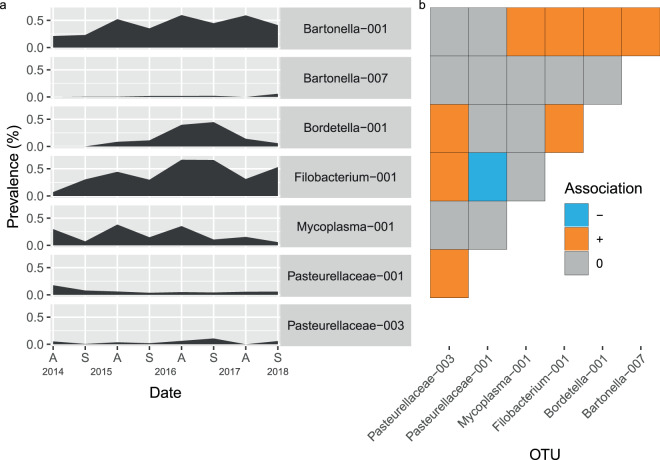
Prevalence and co-occurrence patterns of OTUs detected in spring 2018. (**a**) Prevalences of the 7 OTUs present in spring 2018 animals across all sampling periods (sampling sites considered together). (**b**) Co-occurrence matrix for the 7 OTUs calculated using all animals from all sampling periods and locations. + indicates that these OTUs occurred together in the same animal more than would be expected if they were randomly distributed with respect to each other, − indicates that the pair occurs less frequently than expected, and 0 indicates that the OTUs co-occur as much as would be expected assuming random distribution. Figure prepared in R (version 3.5.2, R Core Team^55^) using packages egg^100^ and ggplot2^101^.

**Figure 10:**
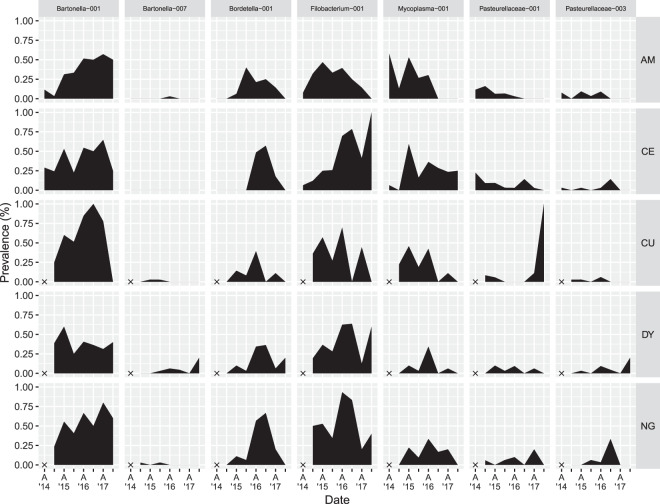
Geographical distribution of OTUs detected in spring 2018. Prevalences of the 7 OTUs present in spring 2018 animals in each sampling period in each sampling location (AM, CE, CU, DY, NG). Periods in which animals were not collected (autumn 2014 in CU, DY, and NG) are indicated with an X. Ticks on the x-axis indicate sampling periods; for legibility, only autumns are labelled. Figure prepared in R (version 3.5.2, R Core Team^55^) using ggplot2^101^.

Seven OTUs were obtained from animals collected in spring 2018, the last sampling period; these 7 OTUs vary in prevalence within the meta-population of *A*. *terrestris* as well as the niches they exploit within the host, but they are similar in that not only are they all found in animals at the end of the outbreak, they are also present in the rest of the sampling sessions with only a few exceptions (Fig. 9a). In addition, all 7 OTUs have a significant association with another OTU in the set; the associations are predominantly positive, that is, the pair is found together more than expected if the OTUs were distributed randomly in the host populations (Fig. 9b). These OTUs are also similarly broadly distributed geographically; with the exception of *Bartonella*-007, these OTUs are detected in at least one sampling period in every site, and frequently they are detected in all locations for a given sampling period (Fig. 10)

## Discussion

We present here the first, to our knowledge, report on the dynamics of the parasitic bacterial communities in the water vole *A*. *terrestris* in relation to the host population dynamics. Using high-throughput sequencing of the V4 region of the 16S rRNA bacterial gene, we have described a diverse and rich community of bacterial parasites that exhibits temporal and spatial heterogeneity over the course of a population outbreak of the rodent host. In addition, we have explored some of the demographic patterns of this rodent host population and have identified several factors that appear correlated with the bacterial parasite community’s dynamics.

Our predictions regarding age structure of the population were not supported: we did not detect a multi-annual aging of the population, changes in age structure were seasonal. This is particularly interesting given that we detected changes in reproductive output; fewer females were pregnant or lactating in spring 2017 relative to spring 2015, but juveniles are clearly being recruited into the trappable population in this period, as evidenced by the lens weights obtained in autumn 2017. Thus, we fail to detect any strong evidence for declines in juvenile survival over the course of the outbreak. In addition to being a marked departure from the age structure results reported by Cerqueira *et al*.^35^, who sampled animals in this region, this is also contrary to patterns of juvenile survival reported for some other cycling microtines, including brown lemmings *Lemmus trimucronatus* and varying lemmings *Dicrostonyx groenlandicus*^62^, wood lemmings *Myopus schisticolor*^63^, and California voles *Microtus californicus*^64^. Juvenile survival was identified as being the second most important demographic parameter for driving changes in population growth rate by Oli and Dobson^65^, and was cited as a key piece of supporting evidence for the senescence hypothesis^66^. However, age at sexual maturity in females and length of the breeding season have also been proposed as critical demographic factors driving changes in population growth rates^67,68^. We cannot assess changes in the length of breeding season with our data, but the lower proportion of females lactating or pregnant in spring 2017 may be an indication that breeding started later that year, contributing to the low population growth rates observed between spring and autumn of that year.

The observed peak in male body condition during the peak phase of the outbreak is broadly consistent with the “Chitty effect”^36,37^; the effect was originally described using male weight uncorrected for size, and using this measure, has been documented in other species and is considered a feature of microtines outbreaks^9,69^. Changes in body weight are thought to be due to external (i.e. environmental) conditions rather than genetic changes in the population^70^, and Oli and Dobson^65^ have suggested that the phenomenon may be a product of energy trade-offs, with more energy being diverted to reproduction at the expense of growth during periods of low abundance. Burthe *et al*.^71^ determined that initial growth rates in *Microtus agrestis* do not vary between phases of the cycle and that the phase-related differences in body size were likely due to the length of the growth period: peak-phase animals get bigger because their juvenile growth period is longer.

Changes in female body condition are less clear; although we do detect declines in body condition, these declines are restricted to autumn animals, aren’t detected in all sampling periods, and where they do occur, they are not all synchronized. Work on stress in *A*. *terrestris* suggests that in females the relationship between stress, and by extension body condition, is related to reproductive status. Charbonnel *et al*.^22^ found that fecal corticosterone metabolite (FCM) concentrations in females collected from late-peak phase populations of *A*. *terrestris* differed between nulliparous females and females that had reproduced, with nulliparous females having higher concentrations. FCM concentrations were also found to be negatively correlated with body condition. While reproduction and body condition were not compared directly, the correlations between reproductive status and FCM, and FCM and body condition, imply that nulliparous females will have lower body condition than females that have reproduced. We observed that mean body condition and the proportion of females that have reproduced within sampling sessions is weakly positively correlated (i.e. when more females have reproduced, we observe high body condition), thus we can speculate that the stress-reproduction-body condition relationships Charbonnel *et al*. observed is also present in our animals, and the geographical variation in declines in female body condition may be a reflection of local effects acting on these relationships.

The parasite community we have recovered is diverse from a taxonomic perspective, representing 11 families within 5 phyla, but also from an ecological perspective, targeting a range of host tissues and utilizing a variety of mechanisms for transmission. The *Bartonella* OTUs are blood parasites, as are most of the *Mycoplasma* OTUs (they are most similar to already described hemotrophic mycoplasmas, see Supplementary Figs. S8, S9 and S10 for details); together, they represent 46% of the OTU assemblage. The lung parasites (*Filobacterium*, *Bordetella*, and the remaining *Mycoplasma* OTUs) represent an additional 24% of the OTU assemblage. *Bartonella* sp. are transmitted by arthropod vectors^72^, while *Filobacterium* sp. and *Bordetella* are spread by contact and aerosols^73,74^, and *Leptospira* sp. is spread through urine^75^. Despite this ecological variety, we still detected clear patterns in parasite richness at multiple scales, indicating that, either through direct interactions like competition or facilitation, or indirect interactions mediated by host characteristics, these parasites do represent a community of interacting agents.

Some of these bacterial taxa are likely to be very common and have been documented in *A*. *terrestris* or other microtines before. *Bartonella* sp. have been detected in *Arvicola* populations in the UK^76,77^, and *Leptospira* sp. has been found in *Arvicola* sp. in the UK and Germany^77–79^. Although we were unable to find reports of either hemotrophic or non-hemotrophic mycoplasmas or *Ureaplasma* in *Arvicola* sp., they are considered widespread in nature^80^ and have been isolated from wild populations of *Myodes glareolus* in Poland^81^ and *Microtus pennsylvanicus* in North America^82^. *Filobacterium* sp. (formerly Cilia-Associated Respiratory Bacillus, or CAR) was a serious problem in lab populations of rodents prior to the widespread adoption of barrier systems in the 1980s^83^, and more recently has been isolated from wild rats in the United States^84^ and wild ruminants in Italy^85^. *Bordetella bronchiseptica* has been isolated from *Microtus agrestis*^15^ and a range of domestic animals^74^. *Mycobacterium microti* can be found in a range of micro-mammals including mice, voles and shrews^86^. *Rickettsia* have been detected in a range of micro-mammals in Europe, including *Apodemus flavicollis*, *A*. *agrarius*, *Myodes glareolus*, *Microtus agrestis*^87,88^.

The consequences of parasitism by these taxa is ambiguous; Gelling *et al*.^77^ reported no significant differences in body condition between *A*. *terrestris* infected with multiple micro- and macro-parasites and uninfected animals. Burthe *et al*.^89^ found that the appearance of *Mycobacterium tuberculosis* lesions in field voles coincided with declines in body condition but not survival, relative to uninfected animals. Experimental infections of *A*. *terrestris* with *Mycobacterium microti* were ultimately fatal^90^. Soveri *et al*.^15^ reported that *Bordetella* infections were fatal in experimentally immunosuppressed *Microtus agrestis* but mortality in animals that were not immunosuppressed was not reported. Many mycoplasmas have been isolated from both diseased and clinically normal animals, making their influence on host health difficult to assess^80^. In short, though we find that changes in the bacterial parasite community coincide with changes in host population abundance and body condition, inferring causality between these changes is not possible with the data we have presented here.

Part of the within-genus diversity we detected is dependent on the methods and conventions we used to identify parasites; specifically, the Swarm algorithm^49^ we used to form OTUs is markedly more conservative than the more typical method of grouping OTUs based on a 97% similarity cut-off; some of our OTUs differ by only a few base pairs and would have been grouped as a single OTU using a 97% cutoff. This raises the possibility that the diversity we have presented here is in fact inflated, and that some of these OTUs actually represent a single taxon; however, inspection of the reference sequences we used for comparisons suggest that many described species also differ by only one or a a few base pairs, if any, in the 16S rRNA gene (See Supplementary Figs. S1–S12 and Supplementary Tables S1–S11 for details). Other studies targeting bacterial parasites in rodents support the idea that host populations can sustain multiple species or strains of the same genus; Birtles *et al*.^91^ used DNA sequencing of the 16S/23S rRNA intergenic region to identify 12 variants of *Bartonella* sp. in a mixed population of wood mice and bank voles; they could assign these variants to 5 species, 2 of which had not been previously described. In addition, some of the genera we have identified here, like *Anaplasma*, are poorly resolved using just the 16S rRNA gene^92^, and the sequencing of other loci would likely reveal that we are underestimating the diversity present in our data set. Finally, while we screened for chimeras twice in our pipeline (before and after OTU formation), there remains the possibility that some of the diversity presented here is due to remaining chimeras that were not identified as such and removed. However, since we filtered our data to only include OTUs found in both PCR replicates of each DNA extraction, we expect any such chimera-related inflation to be low.

Multiple copies of the 16S rRNA gene may also inflate richness estimates; in a survey of publicly available bacterial genomes, Větrovský and Baldrian^93^ reported divergent 16S rRNA copy numbers in a number of bacterial taxa, including *Yersinia pestis*. Divergent copies within the genomes considered tended to be very similar (mean 99.7%) suggesting that OTU formation using a 97% cutoff or the Swarm algorithm will correctly group within-genome copies together as a single unit, but 14 of the genomes considered contained 16S rRNA copies that differed by more than 3%. Further research into 16S rRNA copy numbers within bacterial taxa, and how this may influence surveys of bacterial communities, is warranted.

Infracommunity richness varied seasonally, with higher richness in the autumns and lower richness in the springs, the exception being infracommunity richness in spring 2017, which is higher than any other spring sampled and is similar to autumn richness. This coincides with both low male body condition and low local- and commune-level abundances from which the populations do not recover over the summer (i.e. the decline phase). That spring infracommunity richness is highest when abundance is low is interesting. Poulin and Morand^34^ have described infracommunity richness as the result of two processes: the rate at which infections are established in the host, and the rate at which infections are cleared from the host. The rate of infection establishment is going to be determined by host density for at least some parasites (i.e. those that exhibit density-dependent transmission) thus at low abundance we would expect that the rate of establishment of infections would be lower because contact rates between hosts decline. However, spring 2017 is also characterized by low body condition in males and in females in some populations. Charbonnel *et al*.^22^ has found that, in addition to FCM concentrations and body condition being negatively correlated in *A*. *terrestris*, FCM concentrations and cell-mediated immune function were also negatively correlated, implying that body condition and cell-mediated immune function are positively correlated. If this is the case, and animals collected in spring 2017 have lower immunocompetence relative to animals collected in other springs, then their ability to prevent infections from being established and their ability to clear infections may be compromised, leading to high rates of infection establishment despite low abundance, and low rates of clearance when infections do occur, generating relatively high infracommunity richness. Similarly, Soveri *et al*.^15^ sampled *Microtus agrestis* and *Myodes glareolus* populations in Finland during a decline and found that parasites, bacterial pathogens, and histopathological indications of disease in declining populations were common; although they didn’t assess immunocompetence, and they did not collect animals from other phases from which to compare, the severity of disease observed in collected animals led them to conclude that disease was an important mortality factor in the *Microtus* population.

Component community richness, assessed either by species-accumulation curves or the extrapolated Chao2 estimator, clearly varies over the course of the cycle, but appears unrelated to the host abundance or host demographic characteristics. Extrapolating richness to a sample size of 37 animals at this scale allows us to compare samples while controlling for sample size, but the Chao2 estimator used this way is not an estimate of the total richness of the parasite community. Dove and Cribb^94^ have suggested that the shape of a species-accumulation curve which uses sampling units rather than detections (animals rather than OTU detections) can reflect the similarity between sampling units; steeper curves indicate less similar infracommunities, with animals from the same population hosting very different communities, while shallower curves indicate that animals host similar infracommunities. Since many of our curves at this scale are relatively “short”, ie. they don’t appear to be approaching the asymptote, the lack of clear trend in extrapolated richness may reflect interactions between component community richness and infracommunity similarity that would require additional analyses to disentangle.

Unlike at the component community scale, considering all five sampling sites as a meta-community does reveal patterns in extrapolated OTU richness. The peak in Chao2 richness in autumn 2014 is stark but this may be due to a smaller sample size (only 2 sites were sampled) and in one site (CE) local- and commune-level abundance are very different. If we focus on Chao2 richness after autumn 2014, we can see clear seasonal patterns and a peak in spring richness in 2017, followed a dramatic decline into spring 2018. This is similar to changes in infracommunity richness at both the local and meta-community scale, and may be an indication that in the decline phase, when individuals appear to be most susceptible to infections (as suggested by high infracommunity richness), they are also susceptible to a wider range of infectious agents.

The 7 OTUs recovered from animals collected in 2018 preserve the functional diversity found in the entire data set, with representatives of *Bartonella* sp., *Bordetella* sp., *Mycoplasma* sp., *Filobacterium* sp., and the *Pasteurellaceae* family. At a regional scale these 7 OTUs exhibit a range of prevalence patterns, with the rare OTUs tending to maintain stable prevalence patterns throughout the sampling period, while others, like Filobacterium-001 and Bordetella-001, increasing in prevalence up until spring 2017, after which prevalence either declines or fluctuates. Bajer *et al*.^81^ reported similar trends in haemoparasite persistence in a Polish population of *Myodes glareolus*; they collected animals from 3 sites in 1998, 2002, 2006, and 2010 and found *Hepatozoan erhardovae* and *Trypanosoma evotomys* present in every sampling session at all of the sites. Prevalences varied, with *H*. *erhardovae* exhibiting a decline in prevalence over the study period, but since samplings were spaced at least 4 years apart, it’s difficult to assess whether this is a real trend or a reflection of intra-annual variation. They also reported *Mycoplasma* sp. and *Bartonella* sp. at prevalences of at least 30% at all sites and all sampling periods, but since they do not identify the taxa below the genus level it’s impossible to determine if there was turnover within these genera or whether the same species were detected each time. Birtles *et al*.^91^ reported 5 species of *Bartonella* in a mixed *M*. *glareolus* and *Apodemus sylvaticus* population in the UK, with some individuals hosting all 5 species and rapid turnover in infections, thus pooling all *Bartonella* species together as a single unit for analysis may mask important changes in the parasite community.

The patterns of co-occurrences between these 7 OTUs may contribute to their temporal and spatial persistence; for example, both Filobacterium-001 and Bordetella-001 are lung parasites and their co-occurrence may be an indication that one or both facilitates the other in establishing and maintaining infections. Telfer *et al*.^95^ reported a variety of interactions between a suite of parasites in *M*. *agrestis* in the UK, including increased duration of infection by *Babesia microti* after infection by *Anaplasma phagocytophilum*, while infection by *B*. *microti* decreased duration of infection by *Bartonella taylorii*. Such interactions, if they are present in our communities, are likely to not only influence population-level patterns of parasite community composition richness, but also individual-level patterns of infracommunity richness.

The widespread geographical distributions of the 7 OTUs may be an indication of the connectivity of the host populations; while dispersal in the fossorial form of *A*. *terrestris* is typically over short distances of less than 100 m^96^, Berthier *et al*.^97^ reported that genetically homogeneous populations of *A*. *terrestris* in Franche-Comté could be found at scales of up to 20 km, and Telfer *et al*.^98^ recorded dispersal distances as high as 5 km in Scottish aquatic populations. Modeling work has suggested that such rare long-distance dispersal events are in fact key for generating the traveling wave dynamics observed in this and other cyclic species^99^. Such rare long-distance dispersal events may also contribute to the patterns we have observed here, resulting in the geographically widespread distributions of the 7 OTUs retained in spring 2018.

The results we have presented here demonstrate that characterizing host population dynamics in terms of demography and abundance are crucial for contextualizing parasite communities. Clearly, host abundance, body condition and reproduction are linked to parasite infracommunity and meta-community richness in *A*. *terrestris* in this region, and sampling animals at a single time point would have obscured this point. In addition, sampling at appropriate geographical scales is also clearly important; the variation in infracommunity richness between locations demonstrates that even at scales where we would expect considerable dispersal-based exchanges in hosts and parasites, we find very local effects. If we wish to gain a complete understanding of how parasite communities interact with outbreaking rodent populations, and determine whether such parasite communities play a role in these outbreaks, then investigations must be conducted at temporally and spatially relevant scales, i.e. multiple times per year over multiple years, and at both local and long-distance dispersal scales for the host(s) under consideration.

## Supplementary information

Supplementary Information.

Supplementary Information2.

## Data Availability

Meta-data for the data set analyzed here can be accessed through the dat@osu platform: Fossorial water vole (*Arvicola terrestris*) population cycles and their phase-associated microbial communities (https://dataosu.obs-besancon.fr/FR-180089013067312017-02-03_Fossorial-water-vole-Arvicola-terrestris.html). The raw sequences (fastq format), and raw and filtered tables of abundance for the eight sequencing runs are available in the Zenodo data repository (10.5281/zenodo.3784637). Seed sequences of the OTUs included in the analysis can be found on GenBank, see Supplementary Table S12 for accession numbers.
