# Supplementary material for: Spatio-temporal trends in richness and persistence of bacterial communities in decline-phase water vole populations
Source: Sci Rep. 2020 Jun 11;10:9506. doi: 10.1038/s41598-020-66107-5 (PMC7290036; doi:10.1038/s41598-020-66107-5)
Supplement: Supplementary file 2 — Supplementary Information2. [file 41598_2020_66107_MOESM2_ESM.pdf]

Supplementary Material

Phylogenetic analyses of the bacterial parasites detected in *A. terrestris* populations in Franche-Comte, France, collected in 2014-2018. Bacterial parasites were detected using high-throughput sequencing of the 16S rRNA bacterial gene extracted from organ tissues of the hosts. OTU formation was conducted using the Swarm algorithm [1]; two clustering iterations are preformed - the first to denoise the data, and the second using the first iteration as seed sequences for grouping. Taxonomic assignment was conducted using blastn+ and the SILVA 16S database [2]. Sequence alignments were performed using the web implementation of Clustal Omega with default parameters [3]. The following comparisons to reference sequences were performed using the seed sequences from the first clustering iteration.

Contents

1 Anaplasma 2
2 Bartonella 3
3 Bordetella 10
4 Borelliela 13
5 Filobacterium 14
6 Leptospira 15
7 Mycobacterium 20
8 Mycoplasma and Ureaplasma 22
9 Pasteurellaceae 28
10 Rickettsia 33
11 Yersinia 34
12 GenBank Accession Numbers 35
13 R Packages 36

1 Anaplasma

Anaplasma-001, the only OTU in this data set assigned to the *Anaplasma* genus, is identical to V4 16S rRNA reference sequences of *Anaplasma phagocytophilum* (formerly *Ehrlichia phagocytophilum*) and several unspecified *Ehrlichia* sp. sequences.

Table S1: Similarity, expressed as a percentage, and coverage (in parentheses) of the OTU Anaplasma-001 compared to a selection of reference strains of *Anaplasma phagocytophilum* and unspecified *Ehrlicia*. Sequences were compared using the blastn search tool.

Reference Sequence	Similarity % (Coverage %)
Ehrlichia sp. - U10873	100 (100)
A. phagocytophilum - U02521	100 (100)
Ehrlichia sp. - U77389	100 (100)
Ehrlichia sp. - AJ242784	100 (100)
Ehrlichia sp. - AJ242783	100 (100)
A. phagocytophilum - HM439430	100 (100)
A. phagocytophilum str. JM - CP006617	100 (100)
A. phagocytophilum str. HZ2 - CP006616	100 (100)
Ehrlichia sp. - AF241532	100 (100)
A. phagocytophilum - GQ412339	100 (100)
A. phagocytophilum str. HZ - CP000235	100 (100)

2 Bartonella

The most prevalent of the Bartonella OTUs, Bartonella-001 (global prevalence of 43%), differed from reference sequences for *B. ancashensis*, *B. australis* Aust/NH1, *B. birtlesii*, *B. clarridgeiae*, and *B. taylorii* by one base pair (<1%). The remaining Bartonella OTU's differed from reference *Bartonella* sequences by as much as 1-6 % (2-14 base pairs) and did not form clear affinities to previously-described *Bartonella* species (Figures S1 and S2).

Table S2: Accession numbers, species, host and country of isolation for reference strains of *Bartonella* sp. used in comparisons.

Species - GenBank Accession	Isolation Source	Country
Bartonella alsatica - AJ002139	*Oryctolagus cuniculus*	France
Bartonella ancashensis CP010401	*Homo sapiens*	Peru
Bartonella australis Aust/NH1 - DQ538394	*Macropus giganteus*	Australia
Bartonella bacilliformis - M65249		
Bartonella birtlesii - AF204274	*Apodemus* sp.	France
Bartonella bovis - AF293391	*Bos* sp.	France
Bartonella clarridgeiae - X89208	*Felis catus*	
Bartonella coopersplainsensis - EU111759	*Rattus leucopus*	Australia
Bartonella doshiae - Z31351	*Microtus agrestis*	UK
Bartonella elizabethae - L01260	*Homo sapiens*	USA
Bartonella grahamii - Z31349	*Myodes glareolus*	UK
Bartonella grahamii - CP001562	*Apodemus sylvaticus*	Sweden
Bartonella henselae - BX897699		
Bartonella koehlera - AF076237	*Felis catus*	USA
Bartonella queenslandensis - EU111754	*Melomys sp.*	Australia
Bartonella quintana - M73228	*Homo sapiens*	USA
Bartonella rattaustraliani - EU111749	*Rattus tunneyi*	Australia
Bartonella schoenbuchensis - AJ278187	*Capreolus capreolus*	Germany
Bartonella sp. - U71322	*Peromyscus leucopus*	USA
Bartonella sp. - JF500559	*Miniopterus schreibersii*	Taiwan
Bartonella sp. AA131HXZ - KJ361606	*Apodemus agrarius*	China
Bartonella sp. CR93HXZ - KJ361625	*Myodes rutilus*	China
Bartonella taylorii - Z31350	*Apodemus* spp.	UK
Bartonella tribocorum - AM260525	*Rattus norvegicus*	France
Bartonella vinsonii subsp. *vinsonii* - L01259	Vole (unspecified)	Canada
Brucella microti - AM392286	*Microtus arvalis*	Czech Republic
Daeguia caeni - EF532794		
Falsochrobactrum ovis - KC254733	*Ovis aries*	
Mycoplana dimorpha - D12786		
Ochrobactrum anthropi - NR_114979	*Homo sapiens*	France
Paenochrobactrum gallinarii - FN391023		Germany
Pseudochrobactrum asaccharolyticum - AM180485	*Homo sapiens*	Sweden
Zoogloea ramigera - D14255		

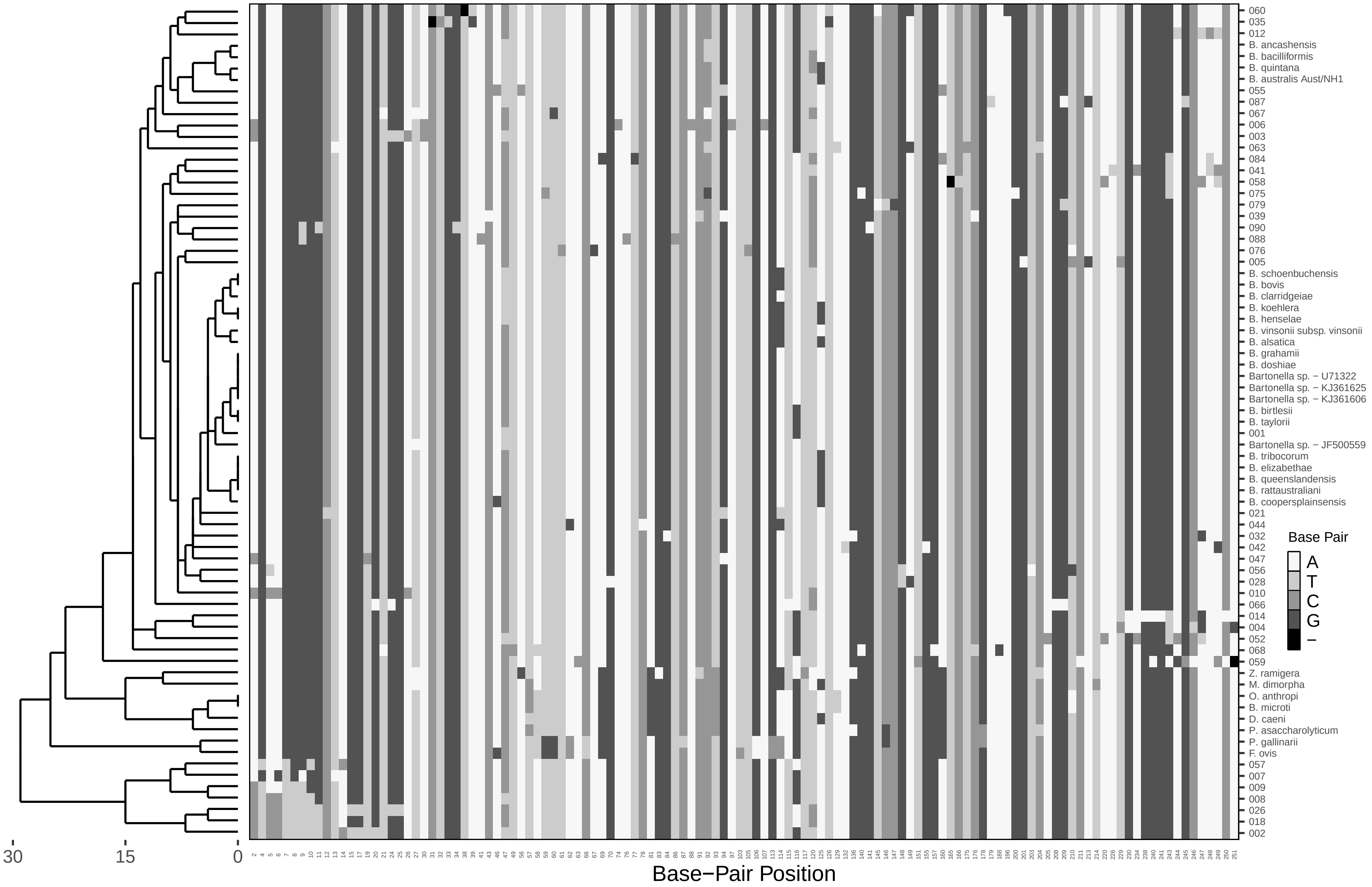
060
035
012
B. ancashensis
B. bacilliformis
B. quintana
B. australis Aust/NH1
055
087
067
006
003
063
084
041
058
075
079
039
090
088
076
005
B. schoenbuchensis
B. bovis
B. clarridgeiae
B. koehlera
B. henselae
B. vinsonii subsp. vinsonii
B. alsatica
B. grahamii
B. doshiae
Bartonella sp. − U71322
Bartonella sp. − KJ361625
Bartonella sp. − KJ361606
B. birtlesii
B. taylorii
001
Bartonella sp. − JF500559
B. tribocorum
B. elizabethae
B. queenslandensis
B. rattaustraliani
B. coopersplainsensis
021
044
032
042
047
056
028
010
066
014
004
052
068
059
Z. ramigera
M. dimorpha
O. anthropi
B. microti
D. caeni
P. asaccharolyticum
P. gallinarii
F. ovis
057
007
009
008
026
018
002
30
15
0
Base−Pair Position
Base Pair
A
T
C
G
−

Figure S1: Variable base-pair positions within the V4 region of the 16S rRNA bacterial gene of a selection of reference *Bartonella* sequences and Bartonella OTUs obtained from *Arvicola terrestris* in Franche-Comté, France. Tile colour indicates the base pair in each sequence at each position. Sequences were clustered using complete linkage of raw distances (ie. no assumptions were made about evolution rates). Black tiles indicate gaps (-) in the alignment

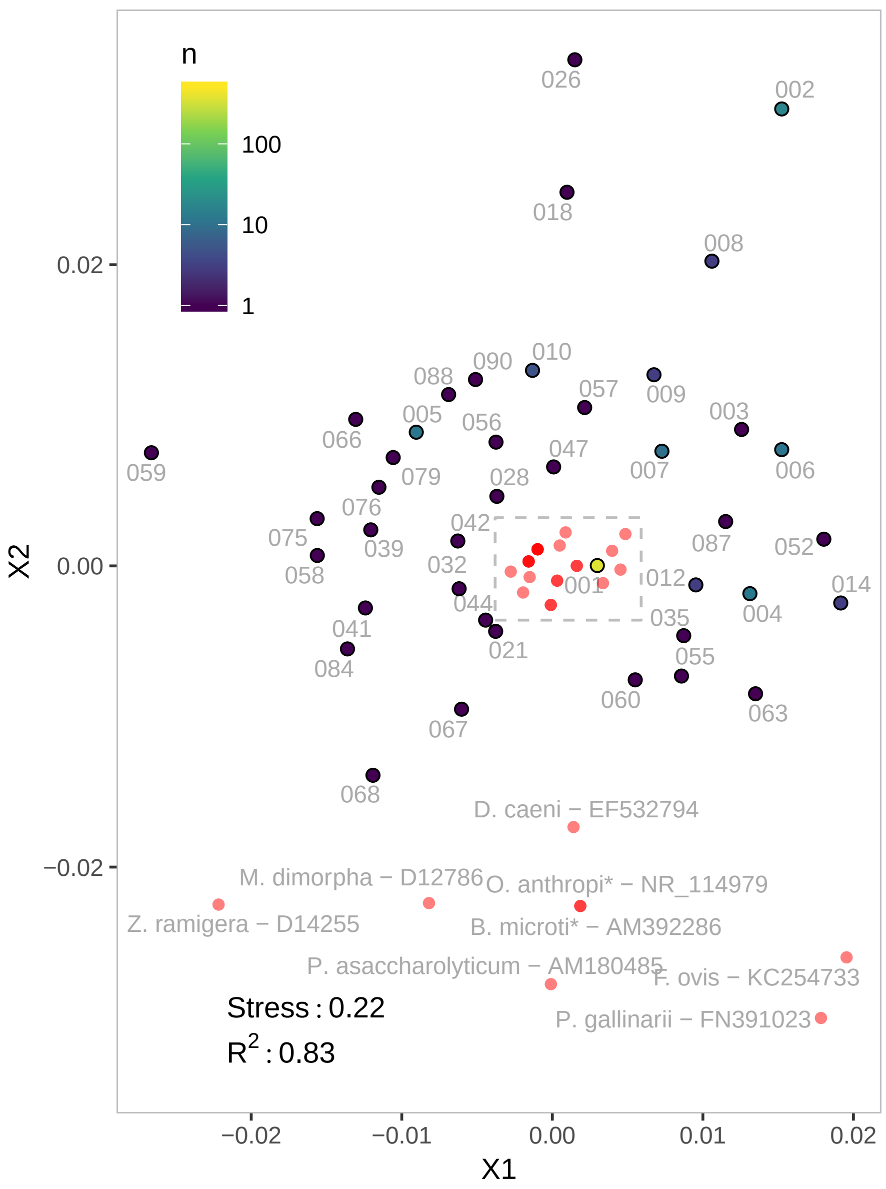
n
100
10
1
026
002
018
008
010
090
088
057
009
005
056
003
066
047
059
079
028
007
006
076
042
075
087
052
039
032
058
001
012
014
044
035
004
041
021
055
084
060
063
067
068
D. caeni − EF532794
M. dimorpha − D12786
O. anthropi* − NR_114979
Z. ramigera − D14255
B. microti* − AM392286
P. asaccharolyticum − AM180485
F. ovis − KC254733
P. gallinarii − FN391023
Stress : 0.22
R2 : 0.83
X2
X1
0.02
0.00
−0.02
−0.01
0.01

Figure S2: nMDS ordination of differences between Bartonella OTU seed sequences (numbered, coloured to indicate frequency of detection within the animals sampled) and reference *Bartonella* sp. sequences (red). Additional reference sequences include *Mycoplana dimporpha*, *Zooglea ramigera*, *Daeguia caeni*, *Falsochromobactrum ovis*, *Paenochrobactrum gallinarii*, *Pseudochrobactrum asaccharolyticum*, *Brucella microti* and *Ochrobactrum anthropi*. See Figure S3 for expanded view of the ordination within the grey dashed box.

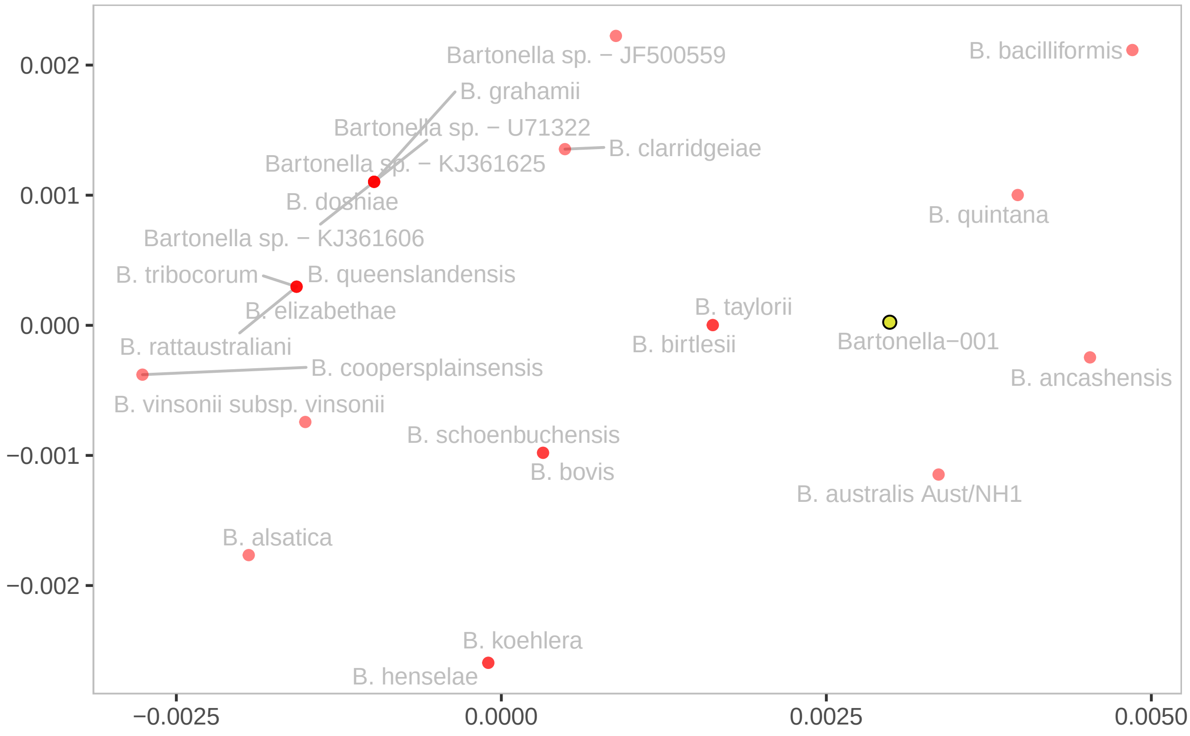

Figure S3: Inset of Figure S2 showing nMDS ordination of differences between the Bartonella-001 seed sequence (yellow) and reference *Bartonella* sp. sequences in red.

3 Bordetella

The seed sequence of Bordetella-001, the most prevalent of the 11 Bordetella OTUs in the data set, is identical to V4 16S rRNA reference sequences of *Bordetella parapertussis* and *B. bronchiseptica*. The seed sequence of Bordetella-003, the second-most prevalent Bordetella OTU, differs from these two reference sequences by 4 base-pairs. The remaining Bordetella OTU seed sequences differ from reference sequences by at least 4 base-pairs and do not show strong affinities with any of the reference sequences.

Table S3: Species and GenBank accession numbers, source and country of isolation for reference strains of *Bordetella* sp. used in comparisons.

Species - GenBank Accession	Isolation Source	Country
B. avium - AF177666	*Meleagris gallopavo*	Germany
B. bronchiseptica - U04948	*Canis lupus familiaris*	
B. hinzii - AF177667	*Gallus gallus*	Australia
B. holmesii - U04820	*Homo sapiens*	USA
B. muralis - LC053647	environment	Japan
B. parapertussis - U04949	*Homo sapiens*	
B. sputigena - KF601914	*Homo sapiens*	Sweden

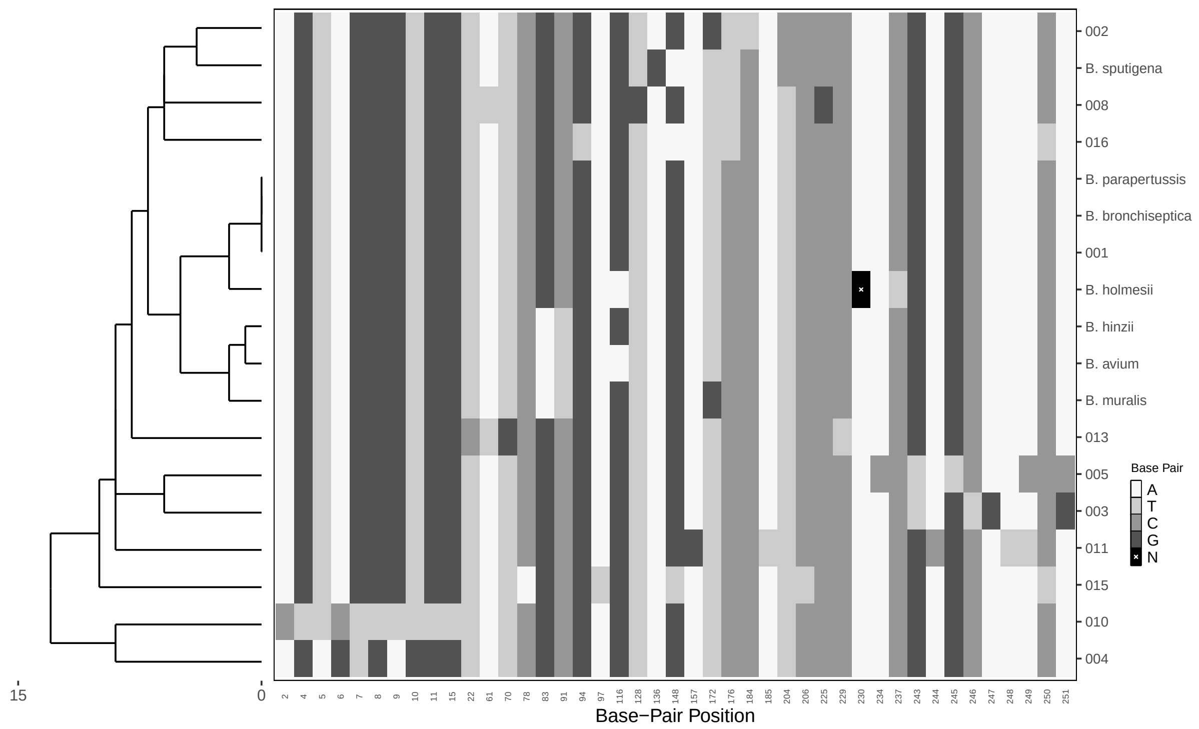

Figure S4: Variable base-pair positions within the V4 region of the 16S rRNA bacterial gene of a selection of reference *Bordetella* sequences and Bordetella OTU seed sequences (numbered) obtained from *Arvicola terrestris* in Franche-Comté, France. Tile colour indicates the base pair in each sequence at each position. Sequences were clustered using complete linkage of raw distances (ie. no assumptions were made about evolution rates). Black crossed tiles (N) indicate ambiguous base pairs in the sequence.

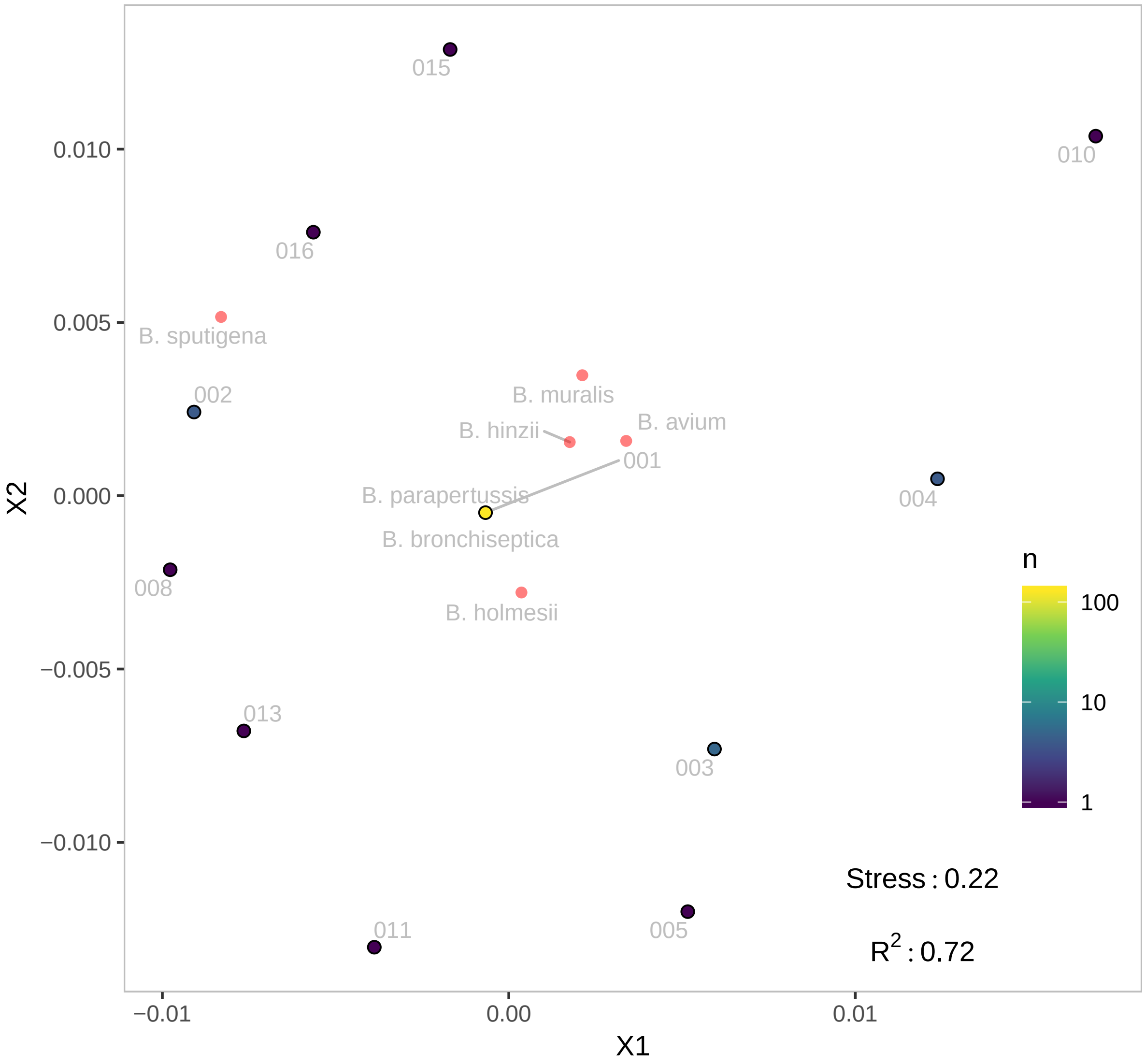

Figure S5: nMDS ordination of differences between Bordetella OTU seed sequences (numbered, coloured to indicate frequency of detection within the animals sampled) and reference *Bordetella* sp. sequences (red).

4 Borelliela

The seed sequence of Borrelliela-001, the only Borrelliela OTU in this data set, is identical to reference sequences of *Borelliella* (*Borellia*) *burgdorferi* as well as an unspecified *Borelliela* strain.

Table S4: Similarity, expressed as a percentage, and coverage (in parentheses) of the OTU Borrelliela-001 compared to a selection of reference strains of *Borreleilla* (*Borrelia*) *burgdorferi* [4]. Sequences were compared using the blastn search tool.

Reference Sequence	Similarity % (Coverage %)
B. burgdorferi (Lyme disease spirochete) - U03396	100 (100)
B. burgdorferi (Lyme disease spirochete) - X85204	100 (100)
B. sp. - AF467971	100 (100)
B. burgdorferi JD1 - CP002312	100 (100)
B. burgdorferi N40 - CP002228	100 (100)
B. burgdorferi ZS7 - CP001205	100 (100)

5 Filobacterium

23 OTUs were assigned to *Filobacterium*; due to the low number of reference sequences available, they were compared to the two reference sequences of *Fiolbacterium rodentium* deposited in GenBank (accession numbers LC036397 and LC055729). The seed sequence of Filobacterium-001 is identical to both reference sequences; the remaining OTU seed sequences range in similarity from 96.8% and 99.6%.

Table S5: Similarity (expressed as a percentage) and coverage of the *Filobacterium* OTU's compared to two reference *Filobacterium* strains from the GenBank database. Sequences were compared using the blast+n search tool during OTU procession

OTUlabel	F. rodentium - LC036397	F. rodentium - LC055729
Filobacterium-001	100 (100)	100 (100)
Filobacterium-002	99.59 (96.8)	99.59 (96.8)
Filobacterium-003	99.58 (94.4)	99.58 (94.4)
Filobacterium-004	97.99 (99.2)	97.99 (99.2)
Filobacterium-006	98.01 (100)	98.01 (100)
Filobacterium-007	97.61 (100)	97.61 (100)
Filobacterium-008	97.61 (100)	97.61 (100)
Filobacterium-009	96.41 (100)	96.41 (100)
Filobacterium-013	98.79 (98.4)	98.79 (98.4)
Filobacterium-017	98.41 (100)	98.41 (100)
Filobacterium-018	98.01 (100)	98.01 (100)
Filobacterium-023	98.39 (98.8)	98.39 (98.8)
Filobacterium-026	98.01 (100)	98.01 (100)
Filobacterium-027	98.41 (100)	98.41 (100)
Filobacterium-029	96.81 (100)	96.81 (100)
Filobacterium-030	97.21 (100)	97.21 (100)
Filobacterium-031	97.61 (100)	97.61 (100)
Filobacterium-032	98.41 (100)	98.41 (100)
Filobacterium-034	97.61 (100)	97.61 (100)
Filobacterium-040	98.01 (100)	98.01 (100)
Filobacterium-043	98.41 (100)	98.41 (100)
Filobacterium-044	98.01 (100)	98.01 (100)
Filobacterium-045	98.01 (100)	98.01 (100)

6 Leptospira

The seed sequence of Leptospira-001, the most globally prevalent Leptospira OTU, is identical to the type sequences for *L. interrogans* and *L. noguchii* Panama. The majority of the remaining Leptospira OTU seed sequences differ from reference sequences by less than 10 base pairs, but few clear affinities between seed sequences and references are present (Figures S6 and S7)

Table S6: Accession numbers, species, host and country of isolation and associated publications for reference strains of *Leptospira* sp. used in comparisons. *Mayotte is a island department of France located in the Indian Ocean

Species - Accession	Isolation Source	Country
Leptospira alexanderi serovar Manhao 3 - AY631880		China
Leptospira alstonii serovar Sichuan - AY631881		
Leptospira broomii - AY796065	*Homo sapiens*	
Leptospira idonii - AB721966		
Leptospira interrogans - Z12817	*Homo sapiens*	
Leptospira kmetyi serovar Malaysia - AB279549		
Leptospira licerasiae serovar Varillal - EF612284	*Homo sapiens*	Peru
Leptospira meyeri serovar Ranarum - AY631878	*Rana pipiens*	USA
Leptospira noguchii serovar Panama - AY631886	*Didelphis marsupialis*	Panama
Leptospira parva serovar Parva - AY293856		
Leptospira santarosai serovar Shermani - AY631883	*Proechimys semispinosus*	Panama
Leptospira wolbachii serovar Codice - AY631879		USA
Leptospira wolffii serovar Khorat - EF025496	*Homo sapiens*	Thailand
Leptospira mayottensis - KJ847187	*Homo sapiens*	France (Mayotte, Indian Ocean)
Leptospira borgpetersenii serovar Tarassovi - AM050577		
Leptospira sp. E156 - LC196101		
Leptospira sp. E152 - LC196099		

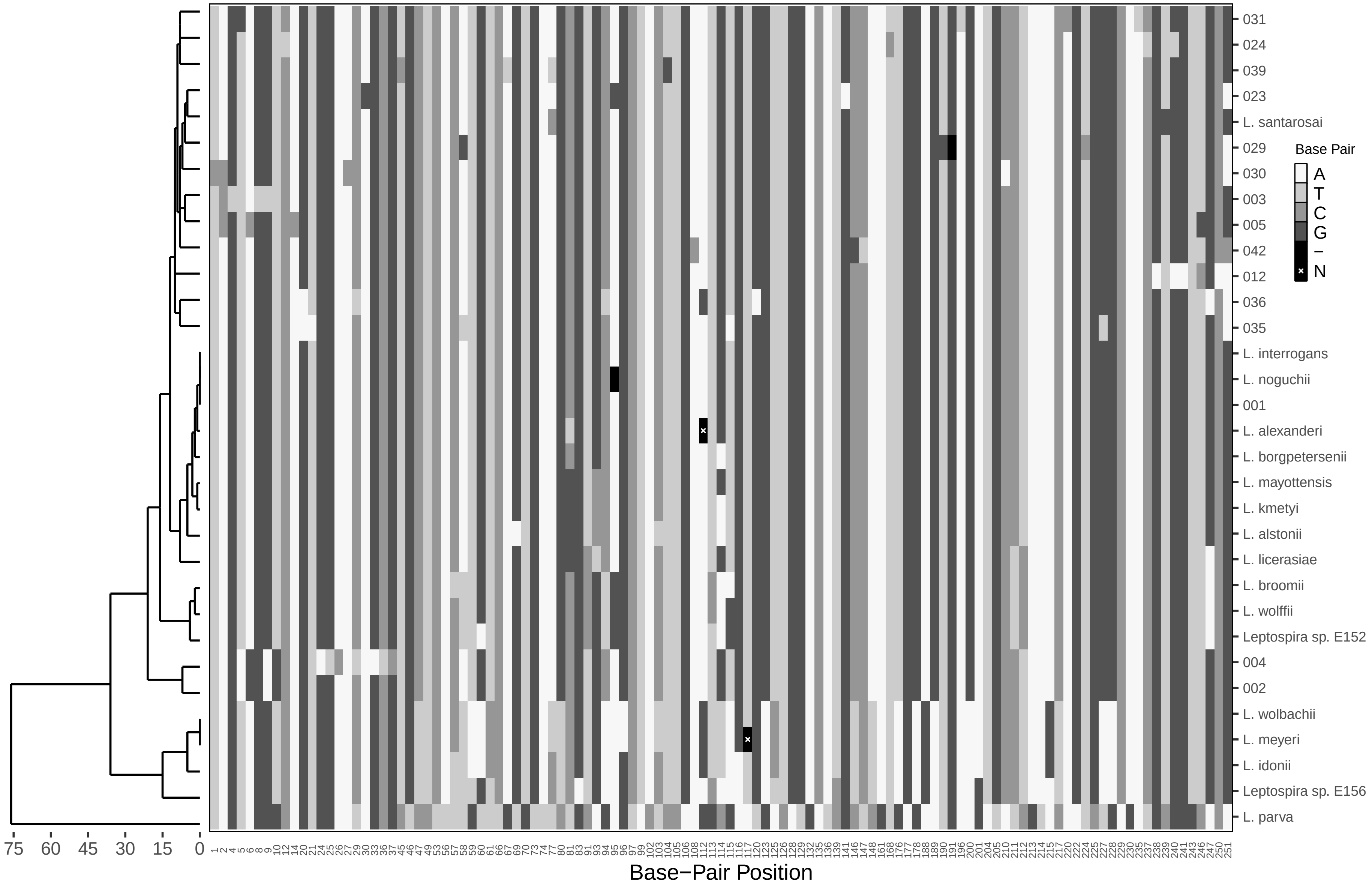
031
024
039
023
L. santarosai
029
030
003
005
042
012
036
035
L. interrogans
L. noguchii
001
L. alexanderi
L. borgpetersenii
L. mayottensis
L. kmetyi
L. alstonii
L. licerasiae
L. broomii
L. wolffii
Leptospira sp. E152
004
002
L. wolbachii
L. meyeri
L. idonii
Leptospira sp. E156
L. parva
Base Pair
A
T
C
G
−
N
75 60 45 30 15 0
Base−Pair Position

Figure S6: Variable base-pair positions within the V4 region of the 16S rRNA bacterial gene of a selection of reference *Leptospira* sequences and Leptospira OTU seed sequences (numbered) obtained from *Arvicola terrestris* in Franche-Comté, France. Tile colour indicates the base pair in each sequence at each position. Sequences were clustered using complete linkage of raw distances (ie. no assumptions were made about evolution rates). Black crossed tiles (N) indicate ambiguous base pairs in the sequence, and black tiles indicate gaps (-) in the alignment

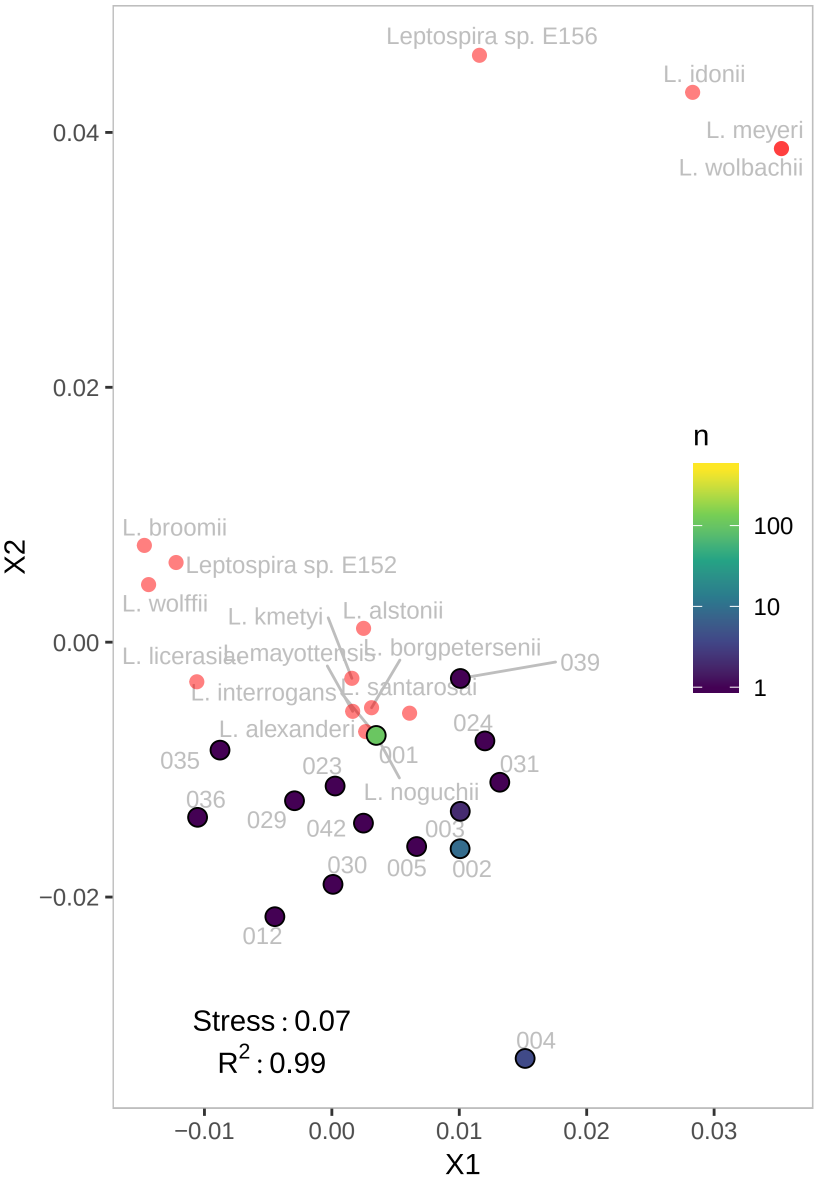

Figure S7: nMDS ordination of differences between Leptospira OTU seed sequences (numbered, coloured to indicate frequency of detection within the animals sampled) and reference *Leptospira* sp. sequences (red).

7 Mycobacterium

The seed sequences of Mycobacterium-001 and -002 are identical to several reference sequences from the *Mycobacterium* genus; Mycobacterium-001 is identical to *M. llatzerense*, *M. mucogenicum*, and *M. phlei*, while Mycobacterium-002 is identical to *M. florentinum*, *M. heidelbergense*, *M. lentiflavum*, *M. kubicae*, *M. montefiorense*, *M. parmense*, *M. stomatepiae*, *M. triplex*.

Table S7: Similarity, expressed as a percentage, and coverage (in parentheses) of the *Mycobacterium* OTU's compared to a selection of typ strains of the genus *Mycobacterium*. Sequences were compared using the blastn search tool.

Reference Sequence	Mycobacterium-001	Mycobacterium-002
M. chelonae - AY457072	99.203 (100)	95.618 (100)
M. florentinum - AJ616230	96.414 (100)	100 (100)
M. heidelbergense - AJ000684	96.414 (100)	100 (100)
M. insubricum - EU605695	100 (100)	96.414 (100)
M. kubicae - AF133902	96.414 (100)	100 (100)
M. lentiflavum - AF480583	96.414 (100)	100 (100)
M. llatzerense - AJ746070	100 (100)	96.414 (100)
M. montefiorense - AF330038	96.414 (100)	100 (100)
M. mucogenicum - AY457074	100 (100)	96.414 (100)
M. paratuberculosis - X52931	96.016 (100)	99.602 (100)
M. parmense - AF466821	96.414 (100)	100 (100)
M. phlei - AF480603	100 (100)	96.414 (100)
M. stomatepiae - AM884331	96.414 (100)	100 (100)
M. triplex - U57632	96.414 (100)	100 (100)
M. tuberculosis - X60070	96.016 (100)	99.602 (100)

8 Mycoplasma and Ureaplasma

The majority of the Mycoplasma OTU seed sequences are most similar to the hemotrophic mycoplasmas, with two matching exactly to reference sequences; Mycoplasma-032 is identical to *M. microti*, and Mycoplasma-002 is identical to an unspecified hemotrophic mycoplasma isolated from *Akodon* sp. (Figures S8 and S9). The remaining hemotrophic mycoplasma OTUs do not form clear affinities to described mycoplasma species, with Mycoplasma-001, the most prevalent, differing from *Mycoplasma haemomurus* by 18 base-pairs. Two OTUs appear more similar to non-hemotrophic mycoplasmas, with Mycoplasma-009 and Mycoplasma-016 most similar to *M. neurolyticum* (differing by 17 and 14 base-pairs respectively).

Table S8: Species and GenBank accession numbers, source and country of isolation for reference strains of *Mycoplasma* sp. used in comparisons.

Species - GenBank Accession	Isolation Source	Country
Anaeroplasma varium - M23934		
Asteroleplasma anaerobium - M22351		
Candidatus *Mycoplasma haemomuris* subsp. *musculi* - AB758440	*Apodemus argenteus*	Japan
Candidatus *Mycoplasma haemomuris* subsp. *ratti* - AB758439	*Rattus rattus*	Japan
Haloplasma contractile - EF999972		
Mesoplasma tabanidae - AY187288	*Tabanus abactor*	USA
Mycoplasma arginini - AF125581	*Panthera leo*	
Mycoplasma arthritidis - M24580	*Rattus* sp.	
Mycoplasma caviae - AF221111	*Cavia porcellus*	
Mycoplasma cavipharyngis - AF125879	*Cavia porcellus*	
Mycoplasma citelli - AF412973	*Citellus richardsonii*	
Mycoplasma coccoides - AY171918	*Mus* sp.	
Mycoplasma collis ATCC 35278 - AF538681	*Rattus* sp.	UK
Mycoplasma cricetuli ATCC 35279 - AF412976	*Cricetulus griseus*	
Mycoplasma haemofelis - AF178677	*Felis catus*	USA
Mycoplasma haemofelis - U88563	*Apodemus argenteus*	Japan
Mycoplasma haemofelis - U95297	*Felis catus*	USA
Mycoplasma haemomuris - U82963	*Apodemus argenteus*	Japan
Mycoplasma microti - AF212859	*Microtus ochrogaster*	USA
Mycoplasma muris - M23939	*Mus* sp.	
Mycoplasma neurolyticum - M23944	*Mus* sp.	
Mycoplasma ovipneumoniae - U44771	*Ovis aries*	
Mycoplasma oxoniensis - AF412987	*Cricetulus griseus*	
Mycoplasma pneumoniae ATCC 15531 - AF132740	*Homo sapiens*	
Mycoplasma pulmonis - AF125582	*Rattus* sp.	
Mycoplasma putrefaciens - U26055	*Capra aegagrus*	USA
Spiroplasma sabaudiense - AY189308	*Aedes sticticus/vexans*	France
uncultured *Mycoplasma* sp. - KT215637	*Akodon* sp.	Brazil (Atlantic Forest)
uncultured *Mycoplasma* sp. - KT215638	*Necromys lasiurus*	Brazil (Atlantic Forest)
Ureaplasma gallorale - U62937	*Gallus gallus domesticus*	
Ureaplasma urealyticum ATCC27618 - AF073450	*Homo sapiens*	

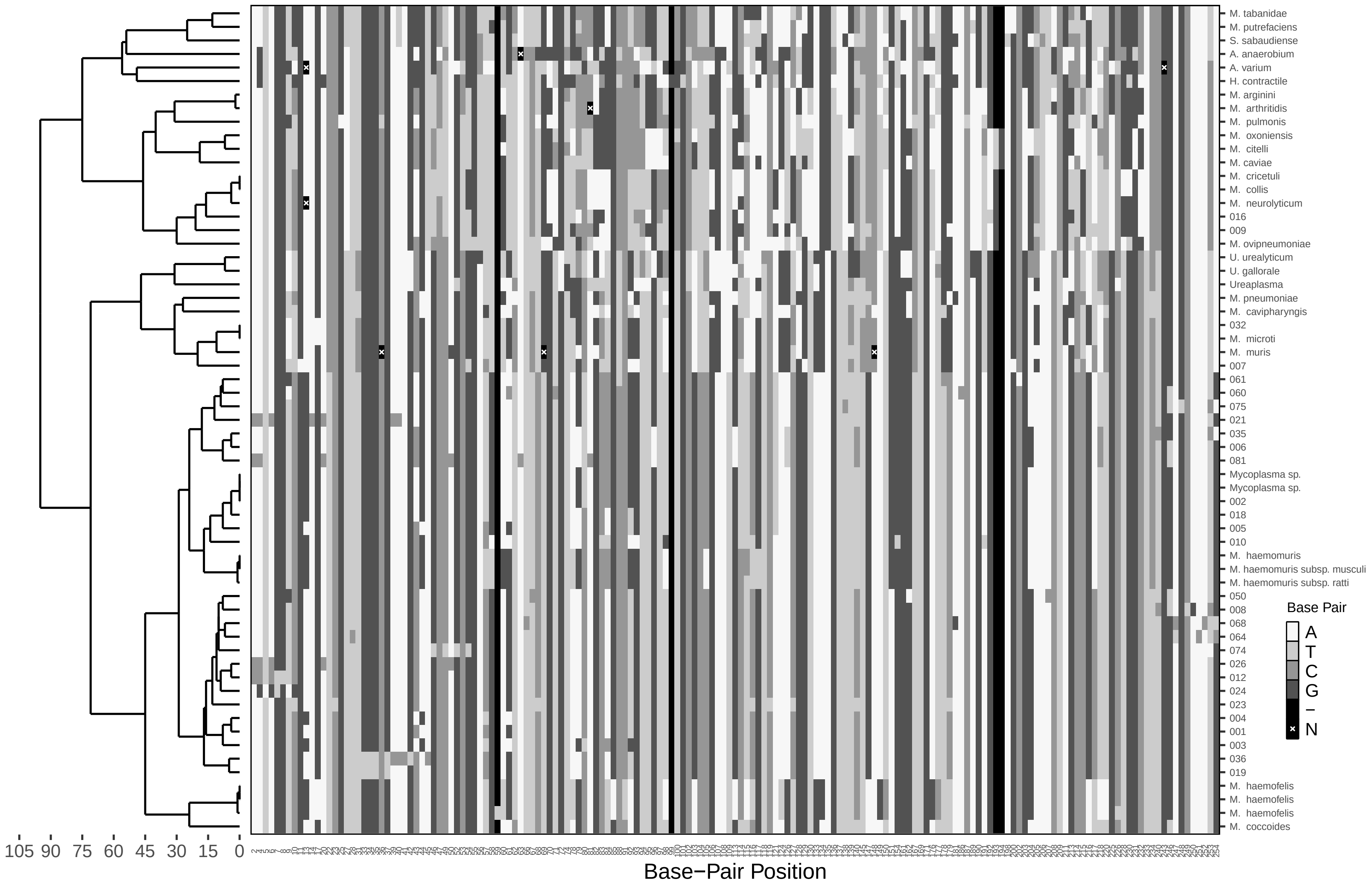

M. tabanidae
M. putrefaciens
S. sabaudiense
A. anaerobium
A. varium
H. contractile
M. arginini
M. arthritidis
M. pulmonis
M. oxoniensis
M. citelli
M. caviae
M. cricetuli
M. collis
M. neurolyticum
016
009
M. ovipneumoniae
U. urealyticum
U. gallorale
Ureaplasma
M. pneumoniae
M. cavipharyngis
032
M. microti
M. muris
007
061
060
075
021
035
006
081
Mycoplasma sp.
Mycoplasma sp.
002
018
005
010
M. haemomuris
M. haemomuris subsp. musculi
M. haemomuris subsp. ratti
050
008
068
064
074
026
012
024
023
004
001
003
036
019
M. haemofelis
M. haemofelis
M. haemofelis
M. coccoides
105 90 75 60 45 30 15 0
Base−Pair Position
Base Pair
A
T
C
G
−
N

Figure S8: Variable base-pair positions within the V4 region of the 16S rRNA bacterial gene of a selection of reference *Mycoplasma* sequences and Mycoplasma OTUs obtained from *Arvicola terrestris* in Franche-Comté, France. Tile colour indicates the base pair in each sequence at each position. Sequences were clustered using complete linkage of raw distances (ie. no assumptions were made about evolution rates). Black crossed tiles (N) indicate ambiguous base pairs in the sequence, solid black tiles indicate gaps (-) in the alignment

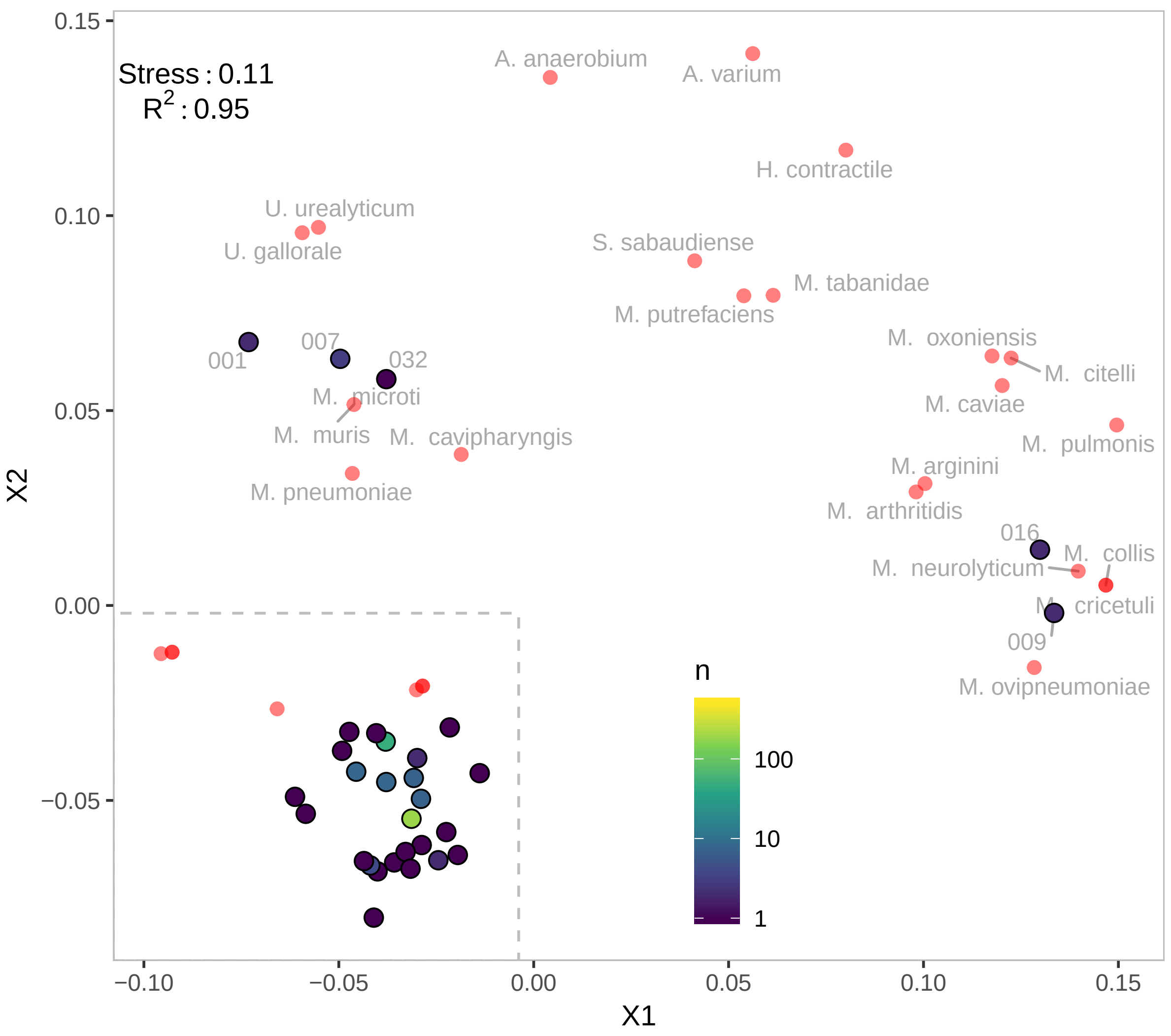

Figure S9: nMDS ordination of differences between Mycoplasma OTU seed sequences (coloured to indicate frequency within the data set) and references Mycoplasma sequences (red). See Figure S10 for expanded view of the ordination within the grey dashed box.

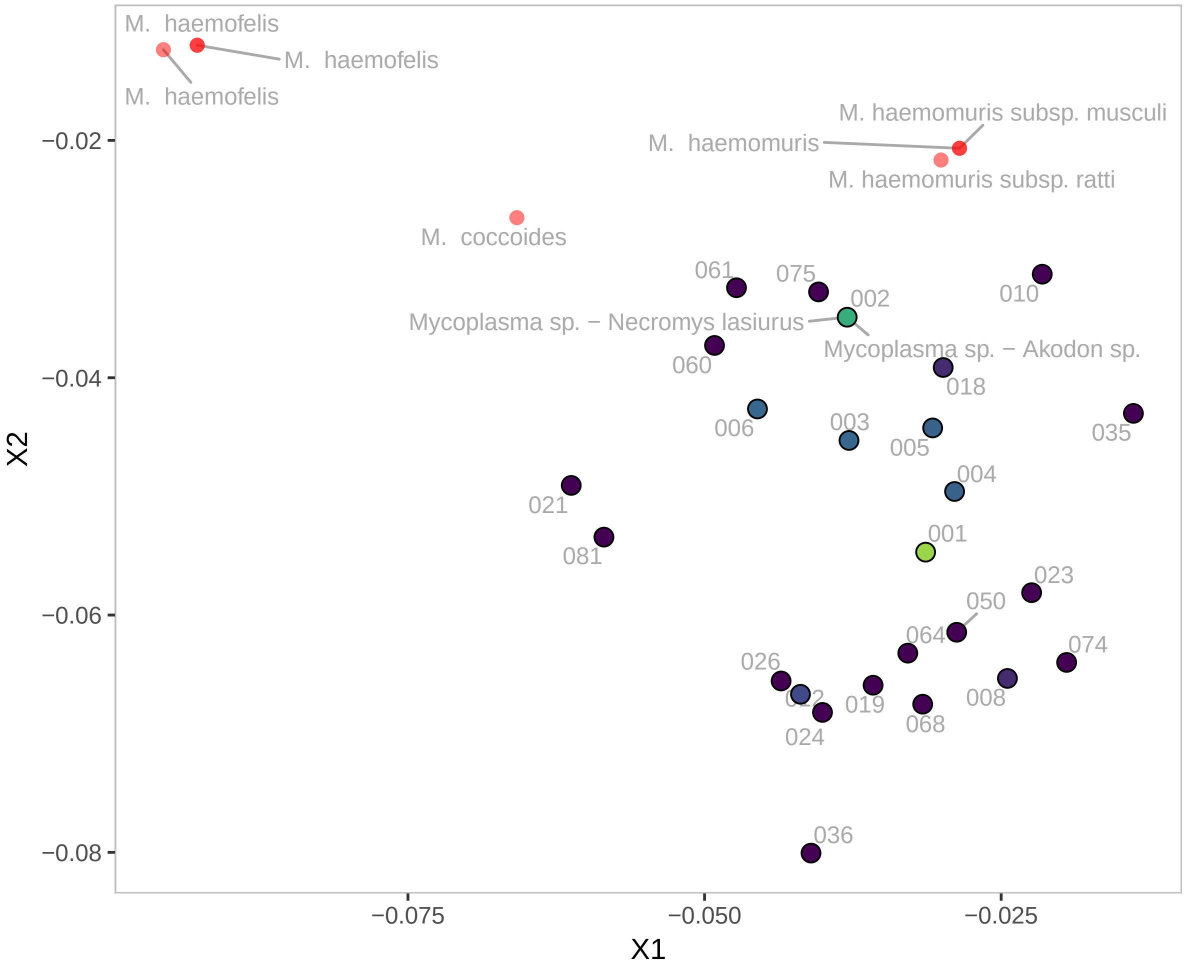

Figure S10: Inset of Figure S9 showing nMDS ordination of differences between Mycoplasma OTU seed sequence (numbered, coloured) and reference *Mycoplasma* sp. sequences in red.

9 Pasteurellaceae

The *Pasteurellaceae* as a family is currently taxonomically ambiguous; with a myriad of undescribed species and strains poorly characterized, new taxonomies are produced relatively frequently, often with radically different topologies depending on the loci under consideration. That being said, we don't attempt to assign genera to the majority of our Pasteurellaceae OTUs unless the seed sequence matches a described species exactly (Actinobacillus-001, for example).

Table S9: Accession numbers, species, host and country of isolation and associated publications for reference strains of *Pasteurellaceae* sp. used in comparisons. *Members of the Rodent Group described in [5].

Species - Accession	Isolation Source	Country
*Muribacter muris** - AF024526	mouse (unspecified)	USA
*Rodentibacter rarus** - AF024529	*Rattus* sp.	USA
*Haemophilus influenzae-murium** - AF024530	mouse (unspecified)	
Mannheimia granulomatis - AF053902	*Bos* sp.	Brazil
Haemophilus felis - AF224292	*Felis catus*	USA
Pasteurella sp. MCCM 00235 * - AF224300		
Pasteurellaceae bacterium* - AF224301	rodent (unspecified)	
*Mesocricetibacter intestinalis** - AF224302	*Mesocricetus auratus*	Germany
*Necropsobacter rosorum** - AF224303	*Cavia porcellus*	Germany
Pasteurella sp. MCCM 02120* - AF224304		
Gallibacterium anatis - AF228001	*Anas platyrhynchos domesticus*	Denmark
Pasteurella multocida subsp. *multocida* - AF294410	*Sus scrofa domesticus*	Canada
Pasteurella multocida subsp. *septica* - AF294411	*Homo sapiens*	France
Pasteurella skyensis - AJ243202	*Salmo salar*	UK
Haemophilus pittmaniae - AJ290755	*Homo sapiens*	Denmark
Haemophilus parahaemolyticus - AJ295746	*Homo sapiens*	USA
Pasteurella multocida subsp. *multocida* - AY078999	*Sus scrofa domesticus*	Canada
Haemophilus aegyptius - AY362905	*Homo sapiens*	USA
Haemophilus haemoglobinophilus - AY362907	*Canis lupus familiaris*	Denmark
Haemophilus parainfluenzae - AY362908	*Homo sapiens*	UK
Glaesserella parasuis - AY362909		
Pasteurella bettyae - AY362917	*Homo sapiens*	UK
Avibacterium gallinarum - AY362921	*Gallus gallus*	Denmark
Pasteurella langaaensis - AY362922		Denmark
Pasteurella mairii - AY362923	*Sus scrofa domesticus*	UK
Bibersteinia trehalosi - AY362927	*Ovis aries*	UK
Avibacterium paragallinarum - AY498868	*Gallus gallus*	Germany
Haemophilus influenzae - M35019	*Homo sapiens*	UK
Haemophilus ducreyi - M63900	*Homo sapiens*	France
Aggregatibacter actinomycetemcomitans - M75039	*Homo sapiens*	UK
Aggregatibacter aphrophilus - M75042	*Homo sapiens*	UK
Aggregatibacter segnis - M75043	*Homo sapiens*	Denmark
Pasteurella oralis - M75052	*Homo sapiens* bitten by *Felis catus*	Germany
Avibacterium avium - M75058	*Gallus gallus*	Germany
Haemophilus paracuniculus - M75061	*Oryctolagus cuniculus*	USA
Avibacterium volantium - M75070	*Galloanserae* sp.	UK
Actinobacillus pleuropneumoniae - M75074	*Sus scrofa domesticus*	Germany
Haemophilus paraphrohaemolyticus - M75076	*Homo sapiens*	UK
Mannheimia haemolytica - M75080	*Ovis aries*	UK
*Rodentibacter pneumotropicus** - M75083	*Mus musculus* (Swiss mouse)	USA
Haemophilus massiliensis - NR149208	*Homo sapiens*	Senegal

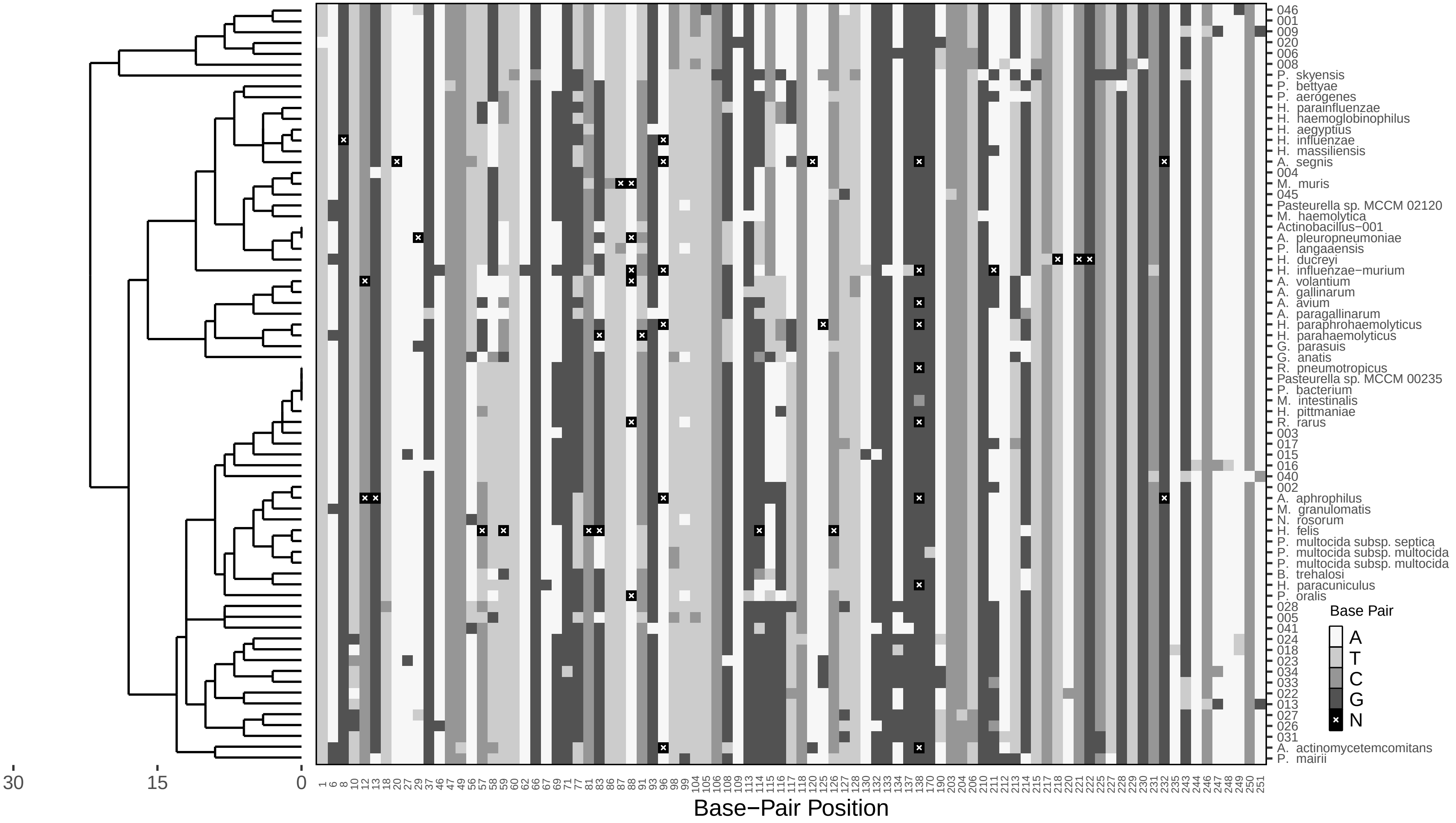

Figure S11: Variable base-pair positions within the V4 region of the 16S rRNA bacterial gene of a selection of reference *Pasteurellaceae* sequences and Pasteurellaceae OTUs obtained from *Arvicola terrestris* in Franche-Comté, France. Tile colour indicates the base pair in each sequence at each position. Sequences were clustered using complete linkage of raw distances (ie. no assumptions were made about evolution rates). Black crossed tiles (N) indicate ambiguous base pairs in the sequence.

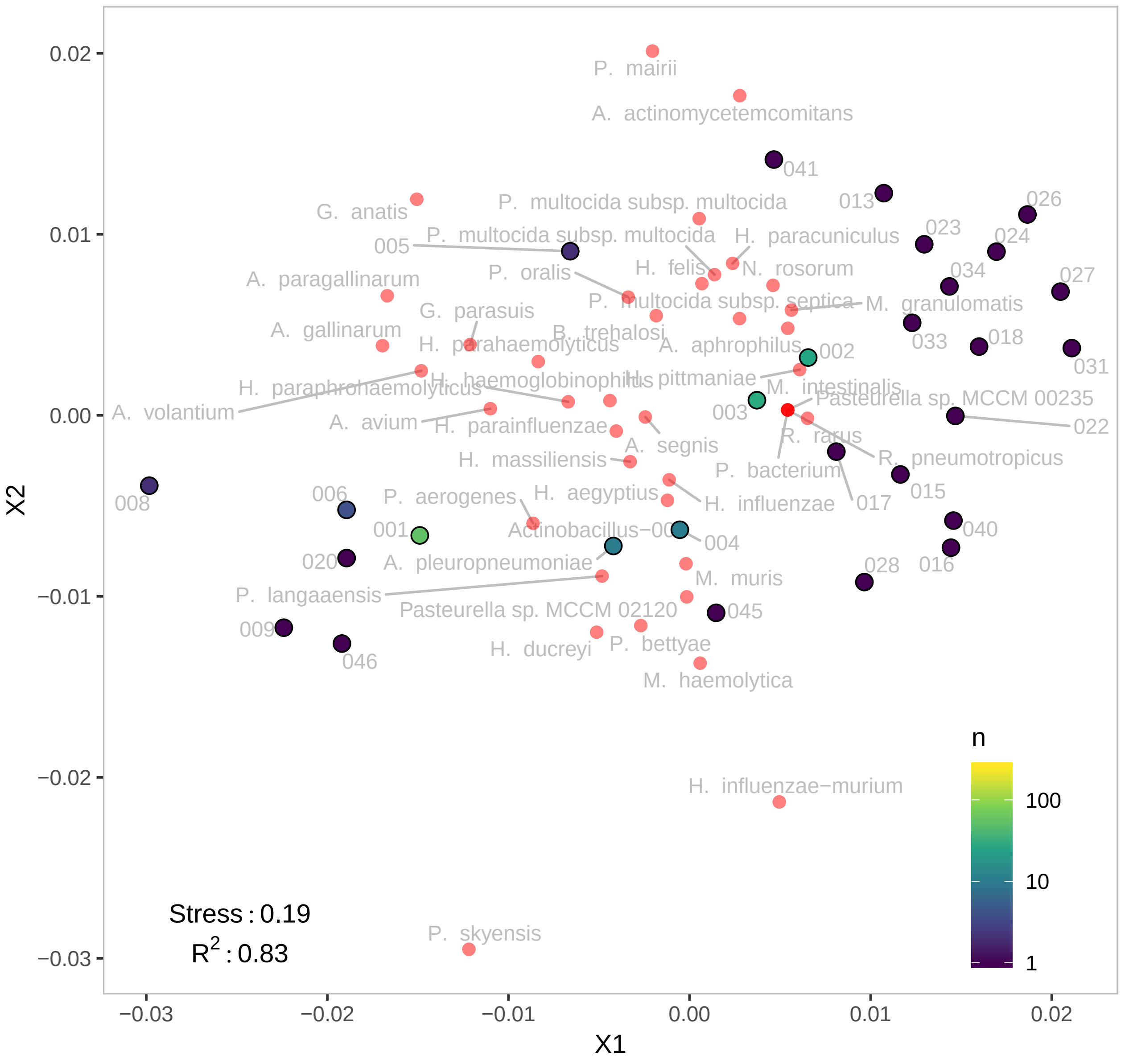
X1
X2
Stress : 0.19
R^2 : 0.83
n
100
10
1
P. mairii
A. actinomycetemcomitans
G. anatis
P. multocida subsp. multocida
P. multocida subsp. multocida
H. paracuniculus
A. paragallinarum
P. oralis
H. felis
N. rosorum
P. multocida subsp. septica
M. granulomatis
G. parasuis
A. gallinarum
B. trehalosi
H. parahaemolyticus
A. aphrophilus
H. paraphrohaemolyticus
H. haemoglobinophilus
H. pittmaniae
M. intestinalis
Pasteurella sp. MCCM 00235
A. volantium
A. avium
H. parainfluenzae
R. rarus
A. segnis
H. massiliensis
R. pneumotropicus
P. bacterium
P. aerogenes
H. aegyptius
H. influenzae
Actinobacillus-00
A. pleuropneumoniae
M. muris
P. langaaensis
Pasteurella sp. MCCM 02120
H. ducreyi
P. bettyae
M. haemolytica
H. influenzae-murium
P. skyensis

Figure S12: nMDS ordination of differences between Pasteurellaceae OTU seed seequences (numbered, coloured to indicate frequency within the data set) and references *Pasteurellaceae* sequences (red).

10 Rickettsia

Only one OTU in this data set was assigned to *Rickettsia*; the seed sequences is most similar to several members of the tick-borne spotted fever group.

Table S10: Similarity, expressed as a percentage, and coverage (in parentheses) of the OTU Rickettsia-001 compared to a selection of reference strains of *Rickettsia* sp. Sequences were compared using the blastn search tool.

OTUlabel	Reference Sequence	Similarity % (Coverage %)
Rickettsia-001	R. canadensis str. CA410 - CP003304	98.01 (100)
Rickettsia-001	R. canadensis - L36104	98.01 (100)
Rickettsia-001	R. rhipicephali - L36216	98.01 (100)
Rickettsia-001	R. canadensis str. McKiel - CP000409	98.01 (100)

11 Yersinia

Only one OTU in this data set was assigned to *Yersinia*; the seed sequence is identical to a number of species in this genus, as well as the type sequence for *Serratia liquefaciens*.

Table S11: Similarity, expressed as a percentage, and coverage (in parentheses) of the Yersinia OTU compared to a selection of reference strains of *Yersinia* sp. and *Serratia liquifaciens*. Sequences were compared using the blastn search tool.

Reference Sequence	Similarity % (Coverage %)
Serratia liquefaciens - CP011303	100 (100)
Y. kristensenii - CP009997	100 (100)
Y. pestis A1122 - CP009840	100 (100)
Y. enterocolitica - CP009838	100 (100)
Y. intermedia - CP009801	100 (100)
Y. aldovae 670-83 - CP009781	100 (100)
Y. rohdei - CP009787	100 (100)
Y. pseudotuberculosis - CP009786	100 (100)

12 GenBank Accession Numbers

Table S12: GenBank accession numbers for OTU seed sequences presented in the text

OTUlabel	accession	OTUlabel	accession	OTUlabel	accession
Actinobacillus-001	MN594323	Borreliella-001	MN594321	Mycoplasma-021	MN594372
Anaplasma-001	MN594320	Filobacterium-001	MN594312	Mycoplasma-023	MN594351
Bartonella-001	MN594311	Filobacterium-002	MN594370	Mycoplasma-024	MN594354
Bartonella-002	MN594344	Filobacterium-003	MN594366	Mycoplasma-026	MN594368
Bartonella-003	MN594343	Filobacterium-004	MN594376	Mycoplasma-032	MN594345
Bartonella-004	MN594337	Filobacterium-006	MN594378	Mycoplasma-035	MN594373
Bartonella-005	MN594338	Filobacterium-007	MN594381	Mycoplasma-036	MN594375
Bartonella-006	MN594347	Filobacterium-008	MN594382	Mycoplasma-050	MN594400
Bartonella-007	MN594341	Filobacterium-009	MN594383	Mycoplasma-060	MN594407
Bartonella-008	MN594355	Filobacterium-013	MN594387	Mycoplasma-061	MN594408
Bartonella-009	MN594356	Filobacterium-017	MN594416	Mycoplasma-064	MN594409
Bartonella-010	MN594350	Filobacterium-018	MN594417	Mycoplasma-068	MN594410
Bartonella-012	MN594339	Filobacterium-023	MN594438	Mycoplasma-074	MN594411
Bartonella-014	MN594353	Filobacterium-026	MN594440	Mycoplasma-075	MN594412
Bartonella-018	MN594364	Filobacterium-027	MN594448	Mycoplasma-081	MN594419
Bartonella-021	MN594380	Filobacterium-029	MN594449	Pasteurellaceae-001	MN594319
Bartonella-026	MN594374	Filobacterium-030	MN594450	Pasteurellaceae-002	MN594317
Bartonella-028	MN594379	Filobacterium-031	MN594451	Pasteurellaceae-003	MN594318
Bartonella-032	MN594385	Filobacterium-032	MN594452	Pasteurellaceae-004	MN594328
Bartonella-035	MN594388	Filobacterium-034	MN594453	Pasteurellaceae-005	MN594358
Bartonella-039	MN594389	Filobacterium-040	MN594454	Pasteurellaceae-006	MN594365
Bartonella-041	MN594390	Filobacterium-043	MN594455	Pasteurellaceae-008	MN594346
Bartonella-042	MN594391	Filobacterium-044	MN594456	Pasteurellaceae-009	MN594357
Bartonella-044	MN594392	Filobacterium-045	MN594457	Pasteurellaceae-013	MN594377
Bartonella-047	MN594398	Leptospira-001	MN594314	Pasteurellaceae-015	MN594386
Bartonella-052	MN594401	Leptospira-002	MN594336	Pasteurellaceae-016	MN594393
Bartonella-055	MN594402	Leptospira-003	MN594367	Pasteurellaceae-017	MN594394
Bartonella-056	MN594404	Leptospira-004	MN594361	Pasteurellaceae-018	MN594403
Bartonella-057	MN594406	Leptospira-005	MN594363	Pasteurellaceae-020	MN594420
Bartonella-058	MN594413	Leptospira-012	MN594371	Pasteurellaceae-022	MN594433
Bartonella-059	MN594414	Leptospira-023	MN594396	Pasteurellaceae-023	MN594434
Bartonella-060	MN594415	Leptospira-024	MN594397	Pasteurellaceae-024	MN594435
Bartonella-063	MN594422	Leptospira-029	MN594418	Pasteurellaceae-026	MN594436
Bartonella-066	MN594423	Leptospira-030	MN594421	Pasteurellaceae-027	MN594437
Bartonella-067	MN594424	Leptospira-031	MN594458	Pasteurellaceae-028	MN594439
Bartonella-068	MN594425	Leptospira-035	MN594462	Pasteurellaceae-031	MN594441
Bartonella-075	MN594426	Leptospira-036	MN594463	Pasteurellaceae-033	MN594442
Bartonella-076	MN594427	Leptospira-039	MN594464	Pasteurellaceae-034	MN594443
Bartonella-079	MN594428	Leptospira-042	MN594465	Pasteurellaceae-040	MN594444
Bartonella-084	MN594429	Mycobacterium-001	MN594325	Pasteurellaceae-041	MN594445
Bartonella-087	MN594430	Mycobacterium-002	MN594329	Pasteurellaceae-045	MN594446
Bartonella-088	MN594431	Mycoplasma-001	MN594313	Pasteurellaceae-046	MN594447
Bartonella-090	MN594432	Mycoplasma-002	MN594315	Rickettsia-001	MN594322
Bordetella-001	MN594316	Mycoplasma-003	MN594332	Ureaplasma-001	MN594330
Bordetella-002	MN594340	Mycoplasma-004	MN594333	Yersinia	MN594327
Bordetella-003	MN594349	Mycoplasma-005	MN594334		
Bordetella-004	MN594359	Mycoplasma-006	MN594324		
Bordetella-005	MN594395	Mycoplasma-007	MN594331		
Bordetella-006	MN594342	Mycoplasma-008	MN594352		
Bordetella-008	MN594384	Mycoplasma-009	MN594326		
Bordetella-010	MN594399	Mycoplasma-010	MN594348		
Bordetella-011	MN594405	Mycoplasma-012	MN594360		
Bordetella-013	MN594459	Mycoplasma-016	MN594335		
Bordetella-015	MN594460	Mycoplasma-018	MN594362		
Bordetella-016	MN594461	Mycoplasma-019	MN594369		

13 R Packages

The following R packages were used in the analyses and preparation of the manuscript:

- **ape** Paradis, E. and Schliep, K. 2018. ape 5.0: an environment for modern phylogenetics and evolutionary analyses in R. – Bioinformatics 35: 526–528
- **cooccur** Griffith, D. M. et al. 2016. cooccur: Probabilistic species co-occurrence analysis in R. – Journal of Statistical Software, Code Snippets 69: 1–17
- **dplyr** Wickham, H. et al. 2020. dplyr: A Grammar of Data Manipulation
- **dunn.test** Dinno, A. 2017. dunn.test: Dunn's Test of Multiple Comparisons Using Rank Sums
- **ecodist** Goslee, S. C. and Urban, D. L. 2007. The ecodist package for dissimilarity-based analysis of ecological data. – Journal of Statistical Software 22: 1–19
- **egg** Auguie, B. 2019. egg: Extensions for 'ggplot2': Custom Geom, Custom Themes, Plot Alignment, Labelled Panels, Symmetric Scales, and Fixed Panel Size
- **emmeans** Lenth, R. 2020. emmeans: Estimated Marginal Means, aka Least-Squares Means
- **english** Fox, J. et al. 2020. english: Translate Integers into English
- **ggdendro** de Vries, A. and Ripley, B. D. 2016. ggdendro: Create Dendrograms and Tree Diagrams Using 'ggplot2'
- **ggplot2** Wickham, H. 2016. ggplot2: Elegant Graphics for Data Analysis. – Springer-Verlag New York
- **ggrepel** Slowikowski, K. 2020. ggrepel: Automatically Position Non-Overlapping Text Labels with 'ggplot2'
- **ggsn** Santos Baquero, O. 2019. ggsn: North Symbols and Scale Bars for Maps Created with 'ggplot2' or 'ggmap'
- **gtable** Wickham, H. and Pedersen, T. L. 2019. gtable: Arrange 'Grobs' in Tables
- **iNEXT** Hsieh, T. C. et al. 2020. iNEXT: Interpolation and Extrapolation for Species Diversity
- **lubridate** Grolemund, G. and Wickham, H. 2011. Dates and times made easy with lubridate. – Journal of Statistical Software 40: 1–25
- **MASS** Venables, W. N. and Ripley, B. D. 2002. Modern Applied Statistics with S. Fourth edn. – Springer, New York
- **multcomp** Hothorn, T. et al. 2008. Simultaneous inference in general parametric models. – Biometrical Journal 50: 346–363
- **plyr** Wickham, H. 2011. The split-apply-combine strategy for data analysis. – Journal of Statistical Software 40: 1–29

purrr Henry, L. and Wickham, H. 2020. purrr: Functional Programming Tools

raster Hijmans, R. J. 2020. raster: Geographic Data Analysis and Modeling

rcompanion Mangiafico, S. 2020. rcompanion: Functions to Support Extension Education Program Evaluation

readr Wickham, H. et al. 2018. readr: Read Rectangular Text Data

rgdal Bivand, R. et al. 2019. rgdal: Bindings for the 'Geospatial' Data Abstraction Library

rgeos Bivand, R. and Rundel, C. 2019. rgeos: Interface to Geometry Engine - Open Source ('GEOS')

sp Bivand, R. S. et al. 2013. Applied spatial data analysis with R, Second edition. – Springer, NY

stplanr Robin Lovelace and Richard Ellison 2018. stplanr: A Package for Transport Planning. – The R Journal 10

stringr Wickham, H. 2019. stringr: Simple, Consistent Wrappers for Common String Operations

tibble Mller, K. and Wickham, H. 2020. tibble: Simple Data Frames

xtable Dahl, D. B. et al. 2019. xtable: Export Tables to LaTeX or HTML

References

[1] Mahé, F., Rognes, T., Quince, C., de Vargas, C., Dunthorn, M.: Swarm: robust and fast clustering method for amplicon-based studies. PeerJ **2**, 593 (2014)

[2] Quast, C., Pruesse, E., Yilmaz, P., Gerken, J., Schweer, T., Yarza, P., Peplies, J., Glockner, F.O., Yarza, P.: The SILVA ribosomal RNA gene database project: improved data processing and web-based tools. Nucleic Acids Research **41**, 590–596 (2013). doi:10.1093/nar/gks1219

[3] Madeira, F., Park, Y.M., Lee, J., Buso, N., Gur, T., Madhusoodanan, N., Basutkar, P., Tivey, A.R., Potter, S.C., Finn, R.D., *et al.*: The embl-ebi search and sequence analysis tools apis in 2019. Nucleic acids research **47**(W1), 636–641 (2019)

[4] Adeolu, M., Gupta, R.S.: A phylogenomic and molecular marker based proposal for the division of the genus borrelia into two genera: the emended genus borrelia containing only the members of the relapsing fever borrelia, and the genus borreliella gen. nov. containing the members of the lyme disease borrelia (borrelia burgdorferi sensu lato complex). Antonie Van Leeuwenhoek **105**(6), 1049–1072 (2014)

[5] Olsen, I., Dewhirst, F.E., Paster, B.J., Busse, H.: Pasteurellaceae. In: Whitman, W.B., Rainey, F., Kämpfer, P., Trujillo, M., Chun, J., DeVos, P., Hedlund, B., Dedysh, S. (eds.) Bergey's Manual of Systematics of Archaea and Bacteria, pp. 1–9. John Wily & Sons, Inc. in association with Bergey's Manual Trust,, ??? (2015). doi:10.1002/9781118960608.fbm00230
